# Phosphoproteomics identifies determinants of PAK inhibitor sensitivity in leukaemia cells

**DOI:** 10.1186/s12964-025-02107-0

**Published:** 2025-03-13

**Authors:** Pedro Casado, Santiago Marfa, Marym M. Hadi, Henry Gerdes, Sandra M. Martin-Guerrero, Farideh Miraki-Moud, Vinothini Rajeeve, Pedro R. Cutillas

**Affiliations:** 1 Centre for Cancer Evolution, Barts Cancer Institute, Queen Mary University of London, London, EC1M6BQ UK; 2 Centre for Haemato-Oncology, Barts Cancer Institute, Queen Mary University of London, London, EC1M6BQ UK

**Keywords:** Acute myeloid leukaemia biomarkers, Cancer, Kinase inhibitors, Lysine demethylase PHF2, Machine learning, Target therapy, PAK inhibitors, PF-3758309, Phosphoproteomics, Proteomics

## Abstract

**Background:**

The P21 activated kinases (PAK) are frequently dysregulated in cancer and have central roles in oncogenic signalling, prompting the development of PAK inhibitors (PAKi) as anticancer agents. However, such compounds have not reached clinical use because, at least partially, there is a limited mechanistic understanding of their mode of action. Here, we aimed to characterize functional and molecular responses to PAKi (PF-3758309, FRAX-486 and IPA-3) in multiple acute myeloid leukaemia (AML) models to gain insights on the biochemical pathways affected by these inhibitors in this disease and identify determinants of response in patient samples.

**Methods:**

We mined phosphoproteomic datasets of primary AML, and used proteomics and phosphoproteomics to profile PAKi impact in immortalized (P31/Fuj and MV4-11), and primary AML cells from 8 AML patients. These omics datasets were integrated with gene dependency data to identify which proteins targeted by PAKi are necessary for the proliferation of AML. We studied the effect PAKi on cell cycle progression, proliferation, differentiation and apoptosis. Finally, we used phosphoproteomics data as input for machine learning models that predicted ex vivo response in two independent datasets of primary AML cells (with 36 and 50 cases, respectively) to PF-3758309 and identify markers of response.

**Results:**

We found that PAK1 activation– measured from phosphoproteomics data– was predictive of poor prognosis in primary AML cases. PF-3758309 was the most effective PAKi in reducing proliferation and inducing apoptosis in AML cell lines. In cell lines and primary cells, PF-3758309 inhibited PAK, AMPK and PKCA activities, reduced c-MYC transcriptional activity and the expression of ribosomal proteins, and targeted the FLT3 pathway in FLT3-ITD mutated cells. In primary cells, PF-3758309 reduced STAT5 phosphorylation at Tyr699. Functionally, PF-3758309 reduced cell-growth, induced apoptosis, blocked cell cycle progression and promoted differentiation in a model-dependent manner. ML modelling accurately classified primary AML samples as sensitive or resistant to PF-3758309 ex vivo treatment, and highlighted PHF2 phosphorylation at Ser705 as a robust response biomarker.

**Conclusions:**

In summary, our data define the proteomic, molecular and functional responses of primary and immortalised AML cells to PF-3758309 and suggest a route to personalise AML treatments based on PAK inhibitors.

**Supplementary Information:**

The online version contains supplementary material available at 10.1186/s12964-025-02107-0.

## Abstract (es)

Current treatments for acute myeloid leukaemia (AML) are ineffective for most patients. Therefore, new treatments for patients that do not respond to approved drugs or who relapse after initial treatment are needed. A group of proteins, named P21 activated kinases (PAK), are frequently overactivated in AML and compounds that inhibit them are effective in killing AML cells. The identification of patients that will respond to PAK inhibitors would be crucial for the translation of these compounds into the clinic by identifying patients more likely to respond. In this work, we studied how three PAK inhibitors modulate protein phosphorylation and expression in AML cell lines and in cells directly obtained from patients. We focused on PF-3758309 because it was the most effective PAK inhibitor. PF-3758309 inhibited the activities of PAK and other kinases and reduced the phosphorylation and expression of key proteins, like c-MYC, which are known to be important for the proliferation of cancer cells. Mathematical modelling using phosphoproteomics data classified leukaemia cells from AML patients as sensitive or resistant to PF-3758309 treatment and identified the phosphorylation of the lysine demethylase PHF2 at Ser705 as an indicator of whether or not patients may respond to PF-3758309, with potential clinical and diagnostic utility. Overall, our data define the molecular and functional response of AML cells to PAK inhibitors and identify molecular indicators that can be used to predict which patients will respond to these treatments. Our findings can therefore contribute to the translation of PAK inhibitors into clinical use.

## Introduction

Acute Myeloid Leukaemia (AML) is a blood disorder caused by the differentiation impairment and clonal expansion of myeloid progenitor cells that ultimately result in bone marrow failure [1]. Most newly diagnosed AML patients achieve complete remission after chemotherapy but many of them relapse and die from their disease [2]. New therapeutic approaches based on inhibitors of key oncoproteins in AML, such as FLT3, IDH1/2 and BCL2, are being gradually introduced by healthcare systems worldwide [3], but responses are heterogeneous, and new therapeutic approaches are needed to treat refractory and relapsed patients.

PAKs (P21-activated kinases) are serine/threonine protein kinases positioned at the intersection of multiple signalling pathways required for oncogenesis. Overactive PAKs confer cancer cells with growth signal autonomy, the ability to evade apoptosis, invade and metastasize new tissues and the acquisition of drug resistance [4]. Members of the PAK family are divided into two groups based on the sequence and structure homology: group I PAKs that includes PAK1, PAK2, and PAK3, and group II PAKs that comprises PAK4, PAK5, and PAK6. In AML patients, PAK1, PAK3, and PAK5 expression have been associated with poor prognosis and PAK2 with favourable prognosis. Mechanistically, PAK1 activity maintains the c-MYC transcriptional program and, in FLT3-ITD and KIT D816V mutated cells, promotes STAT5 nuclear translocation and the transcription of the antiapoptotic protein BCL-XL [5–7].

The relevance of PAK signalling in cancer and other diseases has prompted the development of multiple PAK inhibitors (PAKi) with different mechanisms and selectivity over PAK members. PF-3758309 is an ATP competitive inhibitor firstly described by Murray et al. and developed to target PAK4 and other PAK group members [8]. However, cell free based assays showed that this compound could also efficiently inhibit AMPK, RS6PKs, PKCs, CHEK2 and multiple tyrosine kinases [8]. FRAX-486 is another ATP competitive inhibitor developed by Doland et al. that targets group 1 PAKs with high selectivity over the group 2 member PAK4 [9]. IPA-3 is an allosteric inhibitor developed by et Deacon al. that targets the activation of PAK members of group 1 [10].

Using a bioinformatic approach to infer kinase activity from phosphoproteomics data, we found that PAK is one of the most frequently increased protein kinase activities in primary AML cells [11]. Consistent with this observation, PAK inhibitors have shown to suppress leukaemia growth in vitro and in vivo and induce monocytic differentiation and apoptosis in AML cells [5, 6, 12]. However, these promising results have not been translated into new therapies for the treatment of AML patients. This could partially be due to a lack of mechanistic understanding on how these compounds work at the molecular level, and on a lack of knowledge of the patient population more likely to respond to such treatments.

In this work, we used a phosphoproteomics and proteomics approach coupled to our bioinformatics workflow to determine the effects of the PAKi PF-3758309, FRAX-486 and IPA-3 at the molecular level in AML cell lines and primary cells. We found that PF-3758309 was the most effective drugs in reducing cell proliferation and inducing apoptosis in AML cell lines. This compound inhibited PAK, AMPK, PKCA activities across all models. Functionally, PF-3758309 reduced cell-growth, induced apoptosis, blocked the cell cycle progression and promoted differentiation in AML cell lines.

Since a successful therapy for AML patients based on PF-3758309 would require the identification of patients sensitive and resistant to treatment with this compound. We performed a machine learning (ML) modelling approach based on phosphoproteomics data that accurately classified primary AML cells grown ex vivo as sensitive or resistant to PF-3758309 treatment. Phosphorylation of PHF2 at Ser^705^ was the greater contributor to the accuracy of the ML model of the response to PF-3758309 and correlated with drug response in two independent patient cohorts. Therefore, this phosphorylation site could be used as a response marker for PF-3758309 in AML primary cells.

In summary, our results show that PF-3758309 targeted multiple proteins and biological processes relevant in AML cells. Additionally, we implemented a ML modelling that efficiently classified AML primary cells as resistant or sensitive to PF-3758309 treatment and identified phosphopeptides that could be used as response markers. Therefore, our data and research approach could contribute to the implementation of precision medicine in AML using multi-targeted kinase inhibitors.

## Experimental procedures

### Culture of cell lines

P31/Fuj (JCRB, Cat. # JCRB0091), MV4-11 (ATCC, Cat. # CRL-9591), MOLM-13 (DSMZ, Cat. # ACC 554), THP-1 (DSMZ, Cat. # ACC 16), HEL (DSMZ, Cat. # ACC 11), Kasumi-1 (DSMZ, Cat. # ACC 220), CMK (DSMZ, Cat. # ACC 392), KG-1 (DSMZ, Cat # ACC 14) and CTS [13] cell lines were maintained at a concentration of 0.5-1.5 × 10^6^ cells / mL in RPMI medium supplemented with 10% FBS (Thermo-Fisher Scientific, Cat. # 10500-064) and 1% Penicillin/Ampicillin solution (Thermo-Fisher Scientific, Cat. # 15140122) in an incubator at 37^o^C and 5% CO_2_. For inhibitor treatment, PF-3758309 (Selleckchem, Cat. # S7094), FRAX-486 (Tocris, Cat. # 5190), IPA-3 (Tocris, Cat. # 3622) and navitoclax (Selleckchem, Cat. # S1001) were diluted in DMSO. Vehicle (DMSO) or the indicated concentration of inhibitor were added. Final concentration of DMSO was kept at 0.1% in all conditions.

### Culture of AML primary cells

Primary cells were thawed at 37 °C, transferred to 50 mL falcon tubes and incubated for 5 min at 37 °C with 500 µL of DNAse Solution (Sigma Aldrich, Cat. # D4513-1VL; resuspended in 10 mL of PBS). 10 mL of 2% FBS in PBS were added and cell suspensions were centrifuged at 525 g for 5 min at RT. Supernatants were discarded and cell pellets were resuspended in 10 mL IMDM (Thermo-Fisher Scientific, Cat. # 12440053) supplemented with 10% FBS and 1% Penicillin/Streptomycin, filtered through a 70 μm strainer and counted. 15 × 10^6^ cells in 10 mL of complete IMDM for each sample were treated with vehicle (DMSO) or the indicated concentrations of PF-3758309 for 2 h in an incubator at 37 °C and 5% CO_2_. Final concentration of DMSO was kept at 0.1% for all conditions. Cells were harvested by centrifugation for 5 min at 525 xg at 4 °C, pellets were washed twice with PBS supplemented with phosphatase inhibitors (1 mM Na_3_VO_4_ and 1 mM NaF). Pellets were transferred to low protein binding tubes (Sigma-Aldrich, Cat. # Z666513-100EA) and stored at -80 °C.

### Cell viability and proliferation

Cells were seeded in T25 flask at a density of 0.5 × 10^6^ cells/mL. The number of viable cells was counted at 0, 24, 48 and 72 h after seeding using a Vi-CELL cell counter (Beckman Coulter, Inc., Brea, CA, USA). At least 3 independent biological replicates per condition were performed.

### Apoptosis assays

Cells were seeded in 96-well plates (3 × 10^4^ cells per well in 150 µL) and treated with vehicle (DMSO) or 1 to 10,000 nM of PF-3758309 or FRAX-486 or 200 to 50,000 nM of IPA-3 for 72 h. Final concentration of DMSO was kept at 0.1% for PF-3758309, FRAX-486 and 200 to 20,000 nM IPA-3. For 50,000 nM IPA-3 DMSO was kept at 0.25%. Cells were stained with Guava Nexin reagent (Cytek, Cat. # 4500 − 0453) according to the manufacturer’s instructions. Samples were measured in a Guava PCA cell analyser (Guava Technologies Inc) and data was analysed using CytoSoft (v2.5.7). At least 3 independent biological replicates per condition were performed. Nexin positive cells were considered as apoptotic cells.

### Differentiation assays

Cell differentiation was measured by Fluorescence-activated cell sorter (FACS) analysis using antibodies directed against human CD11b (BD, Cat. # 550019) and CD15 (BD, Cat. # 555402). AML cells were seeded in 6 well plates (0.75 × 10^6^ cell/mL), left overnight and treated with the indicated drugs for 48 h. 1 × 10^6^ cells were transferred to FACS vials (2 vials per condition). After removal of the medium by centrifugation, 50 µL of 2% HAG (human γ-Globulins) were added to the cells and incubated at 4 °C for 20 min. Antibodies (CD11b and CD15) or control isotypes (APC and PE) were added to each different vial per condition and left in the dark for 30 min. To remove antibody excess, cells were washed with 4 mL of 2% FBS in PBS. Then, cell pellets were resuspended in 300 µL of 2% FBS in PBS with DAPI (1/2000) and analysed in a FACS instrument (Fortessa). FACS data were processed using Flow Jo (v10.6.1). Gates were set up to exclude doublets, non-viable cells and debris.

### Western blot analysis

Cells were harvested by centrifugation at 500 x g for 5 min at 4^o^C and cell pellets were washed twice with 1 mL of ice-cold PBS supplemented with 1mM Na_3_VO_4_ and 1 mM NaF and lysed in 150 µL of cell lysis buffer (50 mM Tris-HCl pH 7.4; 150 mM NaCl; 1 mM EDTA pH 8.0; 1% Triton X-100) supplemented with cOmplete protease inhibitor cocktail (1:100; Roche, Cat. # 11697498001), 1mM Na_3_VO_4_ and 1 mM NaF. Cell lysates were left on ice for 30 min and insoluble material was removed by centrifugation at 13,000 rpm for 15 min at 4 °C. Protein concentration was quantified using BCA. 35 µg of protein per condition were separated in SDS-PAGE using Nu-PAGE 4–12% Bis-Tris Gels (Thermo-Fisher Scientific, Cat. # NP0321BOX) and transferred to PVDF membranes. Membranes were blocked for 1 h at RT in 5% milk in TBS-T solution (0.1% Tween 20 in TBS), probed with primary antibodies overnight at 4 °C, washed with TBS-T solution, probed with secondary antibodies conjugated with horse radish peroxidase (HRP) for 1 h at RT and washed with TBS-T solution. Protein bands were visualized using enhanced chemiluminescence (ECL). Antibodies against c-MYC (Cell Signaling Technologies, Cat. #5605), pFLT3 at Tyr^591^ (Cell Signaling Technologies, Cat. # 3466), pSTAT5A and Tyr^694^ (Cell Signaling Technologies, Cat. # 9351) and Cleaved PARP (Cell Signaling Technologies, Cat. # 9541), were used at a concentration of 1:1,000, antibodies against α-tubulin (Cell Signalling Technologies, Cat. #2144) and vinculin (Sigma-Aldrich, Cat. #V9131) were used at 1:10,000 and antibodies against GAPDH (Abcam, Cat. # Ab9485), rabbit (GE Healthcare, Cat. #NA934V) and mouse (GE Healthcare Cat. #NXA901) IgG were used at 1:5,000.

### Cell lysis for mass spectrometry and trypsin digestion

Cells were harvested by centrifugation at 500 xg at 4^o^C for 5 min, washed twice with cold PBS supplemented with 1mM Na_3_VO_4_ and 1 mM NaF, snap frozen and stored at -80^o^C until further processing. Cell pellets were lysed in urea buffer (8 M urea in 20 mM in HEPES pH 8.0 supplemented with 1 mM Na_3_VO_4_, 1 mM NaF, 1mM Na_4_P_2_O_7_ and 1 mM sodium β-glycerophosphate) for 30 min and further homogenized by sonication (60 cycles of 30s on 40s off; Diagenode Bioruptor^®^ Plus, Liege, Belgium). Insoluble material was removed by centrifugation at 20,000 xg for 10 min at 5^o^C and protein in the cell extracts was quantified by bicinchoninic acid (BCA) analysis. We processed the protein extracts following published methods [14] with some modifications. Briefly, 200 µg and 100 µg of protein were used for phosphoproteomics and proteomics experiments, respectively. Proteins were reduced and alkylated by sequential incubation with 10 mM DTT and 16.6 mM iodoacetamyde for 1 h. The urea concentration was diluted to 2 M with 20 mM HEPES (pH 8.0) and 80 µL of conditioned trypsin beads (50% slurry of TLCK-trypsin; Thermo-Fisher Scientific; Cat. #20230) were added. Beads were conditioned with 3 washes of 20 mM HEPES (pH 8.0). Samples were incubated for 16 h at 37^o^C with agitation. Trypsin beads were removed by centrifugation at 2,000 xg for 5 min at 5^o^C.

### Desalting

Peptide solutions were desalted using 10 mg OASIS-HLB cartridges (Waters, Manchester, UK). OASIS cartridges were accommodated in a vacuum manifold (-5 mmHg), activated with 1 mL ACN and equilibrated with 1.5 mL washing solution (1% ACN, 0.1% TFA). Samples were loaded in the cartridges and the cartridges were washed with 1 mL of washing solution. For proteomics analysis, peptides were eluted with 500 µL of ACN solution (30% ACN, 0.1% TFA), dried in a speedvac (RVC 2–25, Martin Christ Gefriertrocknungsanlagen GmbH) and stored at -80^o^C. For phosphoproteomics analysis, peptides were eluted with 500 µL of glycolic acid buffer 1 (1 M glycolic acid, 50% ACN, 5% TFA) and subjected to phosphoenrichment.

### Phosphoenrichment

Phosphopeptides were enriched using TiO_2_ (GL Sciences) as previously described with some modifications [15]. Sample volumes were normalized to 1 mL using glycolic acid buffer 2 (1 M glycolic acid, 80% ACN, 5% TFA) and 50 µL of TiO_2_ beads (50% slurry in 1% TFA) were added to the peptide mixture. Samples were incubated in a rotator for 5 min at room temperature, centrifuged for 30s at 1,500 xg and 80% of the supernatant was transferred to fresh tubes and stored in ice. Bead pellets were resuspended in the remaining 20% of the supernatant, loaded into empty prewashed PE-filtered spin-tips (Glygen, MD, USA) and packed by centrifugation at 1,500 xg for 3 min. The remaining volume of the supernatant was loaded by centrifugation at 1,500 xg for 3 min and spin tips were sequentially washed with 100 µL of glycolic acid buffer 2, ammonium acetate buffer (100 mM ammonium acetate in 25% ACN) and 10% ACN by centrifugation for 3 min at 1,500 xg. Phosphopeptides were eluted 4 times by adding 50 µL of 5% ammonium water and the centrifuging for 5 min at 1,500 xg. Eluents were snap frozen in dry ice, dried in a speedvac and peptide pellets stored at -80^o^C.

### Acquisition of mass spectrometry

Samples were run in the LC-MS/MS system as previously described [16]. Phosphopeptide pellets from primary samples were resuspended in 20 µL of reconstitution buffer 1 (3% ACN; 0.1% TFA) and run in a Dionex UltiMate 3000 RSLC nano coupled to an Orbitrap Q Exactive Plus mass spectrometer (Thermo Fisher Scientific). Peptide separation in the LC system was performed using Solvent A (0.1% FA) and solvent B (100% + 0.1% ACN) as mobile phases. First, 4 µL of reconstituted phosphopeptide solution were loaded in a µ-precolumn (Cat. # 160454) for 2 min at a back pressure of 15 bar and a nanoflow of 10 µL/min. Then, phosphopeptides were separated in an Acclaim PepMap 100 column (Cat # 164569) using a gradient that went from 3 to 23% B during 120 min with a background pressure of 155 bar and a nanoflow of 0.3 µL/min. Finally, the column was washed with 23–85% B for 7 min and equilibrated with 3% B for 3 min. Peptides were directly transferred to the Q Exactive Plus system where full scan survey spectra (m/z 375-1,500) were acquired with a 70,000 resolution. The 20 most intense ions for each full MS scan were selected for higher energy collisional dissociation (HCD) and MS/MS scanning (200-2,000 m/z) with a resolution of 17,500. Selection was performed in a data-dependent manner using an isolation width of 1.6 Da and a dynamic exclusion window of 10 ppm mass for 30s. Cell line experiments were run in a nano-flow ultrahigh pressure liquid chromatography (UPLC, nano Acquity, Waters) coupled to an LTQ-Orbitrap XL mass spectrometer (Thermo Fisher Scientific). Phosphoproteomics and proteomics pellets were resuspended in 20 and 200 µL of reconstitution buffer 2 (0.1% TFA), respectively and 4 µL were injected in the LC-MS platform. Solvent A (0.1% FA) and solvent B (100% ACN + 0.1% FA) were used as mobile phases in the LC system. Peptides were loaded in a nano ACQUITY UPLC Symmetry C18 Trap Column (Cat # 186006527) for 8 min at a nano-flow of 2 µL/min. Peptides were separated in a ACQUITY UPLC Peptide BEH C18 nano ACQUITY Column (Cat # 186003543). For peptide elution, the LC system established a gradient from 5 to 35% of solvent B during 150 min with a background pressure of 4,000 psi and a nano flow of 0.3 µL/min. The column was washed with 35–85% B for 10 min and equilibrated with 1% B for 15 min. Peptides were directly transferred to the LTQ-Orbitrap XL mass spectrometer. Full scan survey spectra (m/z 375-1,800) were acquired with 30,000 resolution at m/z 400. The 5 most intense ions in each full MS scan were selected for collision induced dissociation (CID) and MS/MS scanning (50 − 2,000 m/z). The ion selection was performed in a data-dependent manner with a dynamic exclusion list of 30 s for 500 entries and 10 ppm mass window, and the fragmentation was performed at 35% normalized energy collision. The described settings produced instrument duty cycles of 2.1s for phosphoproteomics and 2.5s for proteomics experiments. Therefore, the chromatographic peaks for both types of experiments were about 30 s at the base which allowed the construction of extracted ion chromatograms (XICs) with least 10 data points.

### Peptide identification

Peptide and protein identification from mass spectrometry data was performed as previously described [16]. Mascot Daemon (v2.5.0) automated the peptide and protein identification from MS/MS data. Mascot Distiller (v2.4.3.1) generated peak-list files (MGFs) and Mascot 2.5 search engine matched peaks to peptides comprised in proteins annotated in the SwissProt Database restricted to *Homo sapiens* taxon (uniprot_sprot_2014_08.fasta; 20194 sequences for primary cell samples and SwissProt_2012Oct.fasta for cell line samples) with a FDR of ~ 1%. Two trypsin miss cleavages, the fixed modification of carbamidomethyl Cys and the variable modifications PyroGlu on N-terminal Gln; and oxidation of Met were allowed for the analysis of proteomics data. The analysis of phosphoproteomics data also included the variable modification of phosphorylation on Ser, Thr, and Tyr. A mass tolerance of 10 ppm for the MS scans and 25 mmu or 0.8 Da for the MS/MS scans was permitted for primary cell samples and cell line samples, respectively.

### Peptide and protein quantification

For label-free peptide quantification, Pescal software constructed extracted ion chromatograms (XICs) for all identified peptides across all samples and calculated the area under the peak [17]. The XIC windows were 7 ppm and 2 min. Individual peptide intensity values in each sample were normalized to the sum of the intensity values of all the peptides quantified in that sample. Data points not quantified were given a peptide intensity value equal to the minimum intensity value quantified for that particular peptide across all samples divided by 10. For phosphoproteomics experiments, we obtained a phosphorylation index (ppIndex) by summing the signals of all peptide ions containing the same modification site. Phosphopeptide intensities for primary cells treated with PF-3758309 were given as the average of the intensities of 2 technical replicates. Protein intensity values were calculated by adding the intensities of all the peptides derived from a protein. Protein score values were expressed as the maximum Mascot protein score value obtained across samples.

### Estimation of kinase activity

Kinase activity was estimated using Kinase Substrate Enrichment Analysis (KSEA) using the pSite, Signor and PDTs databases to link kinases and substrates [11, 18] and distance as a metric to calculate differences. The analysis was automated with KSEA Plus (v1.0) [19] that can be downloaded from https://github.com/CutillasLab/KSEA_plus. Enrichment of phosphopeptides targeted by specific inhibitors was calculated using the compound-target activity marker (CTAM) database [20] and was also automated with KSEA Plus (v1.0).

### Estimation of transcription factor activity

Transcription factor activity was estimated based on the protein expression of their associated genes using a script computed in an R environment. Genes were linked to transcription factors using the Omnipath database [21]. The script calculates z-scores across samples for the genes linked to a particular transcription factor and sums all z-scores in the same sample to produce a transcription factor activity score.

### Generation of machine learning models

Machine learning models (ML) to classify AML primary samples as sensitive or resistant to ex vivo treatment with PF-3758309 were generated in an R environment using the “caret” package. We randomly divided our sample cohort in training (*n* = 27) and testing sets (*n* = 9) and used average sensitivity as the threshold to assign cases as being resistant or sensitive (Fig. 8A). To reduce the high variable to sample ratio that could lead to the overfitting of the ML models, we classified samples in the training set as sensitive or resistant using a partial lease squares (LPS) algorithm and, we selected the variables that were more important for the generation of the LPS model (importance > 75) (Fig. 8A). Then, we constructed a random forest (RF) model that classified the training set samples as sensitive or resistant using the variables selected based on their importance for the LPS model. We applied the generated RF model to the testing set and we annotated the predicted probability of each testing set sample of being resistant (Fig. 8A). We repeated this modelling procedure 50 times each time selecting a different set of samples for training. To estimate the ability of our models to differentiate between sensitive and resistant samples, we calculated sensitivity and specificity and plot these parameters in a receptor to operator curve (ROC).

### Experimental design and statistical rationale

The number of technical replicates and independent experiments is indicated for each experiment in its corresponding figure legend. For phosphoproteomics and proteomics experiments in AML cell lines, independent replicates refer to cells treated and collected in different days. For phosphoproteomics experiments in AML primary cells, each replicate represents cells extracted from a different patient. Calculation of statistical significance and fold change between conditions was automated in R (V 2023.06.2).

Statistical significance between conditions was assessed using unpaired two tailed Student’s t-test for the experiments with cell lines and paired two tailed Student’s t-test for the experiments with primary samples.

Kaplan-Meier curves were constructed in an R environment using the “survival” and “survminer” packages. Log-Rank test was used to assess statistical differences between survival curves.

## Results

### PAK phosphorylation is prognostic in primary AML

**Fig. 1:**
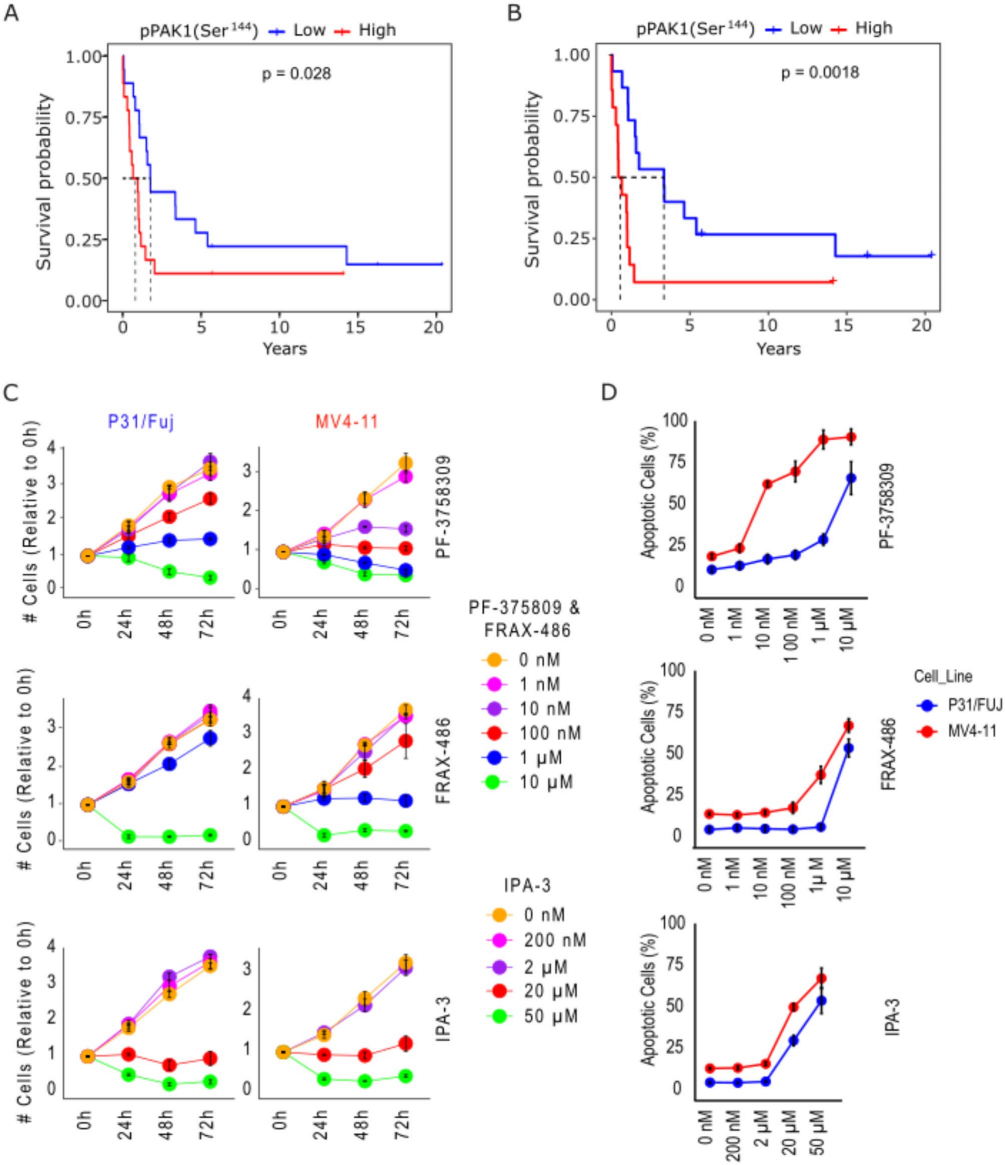
PAK inhibitors as potential treatment for AML. **A**. High PAK1 phosphorylation at Ser^144^ is linked to reduced survival in a cohort of AML patients [14] when considering all patients (*n* = 36) **B**. Link between high PAK1 phosphorylation at Ser^144^ and reduced overall survival in the patient cohort shown in A when only patients younger than 65 years (*n* = 34) are considered. **C**. Effect of the indicated concentrations of PF-3758309, FRAX-486 and IPA-3 on the proliferation of P31/Fuj and MV4-11 cells during a time-course of 72 h. **D**. Apoptosis after 72 h treatment with the indicated concentrations of PF-3758309, FRAX-486 and IPA-3 in P31/Fuj and MV4-11 cells. (*n* > 3 independent experiments). In A and B, Log-Rank test was used to assess statistical differences between survival curves

PAK1 expression has been linked to adverse prognosis in AML [5]. However, whether the phosphorylation and activation of this protein is also prognostic is not known. We mined phosphoproteomics data obtained from a recent study that focused on the analysis of AML cases with poor prognosis [22]. We found that high phosphorylation of PAK1 at Ser^144^– an activatory phosphorylation event [23]– is associated to a shorter overall survival when considering all patients (Fig. 1A) or only patients younger than 65 years (Fig. 1B). Especially, younger patients with high PAK1 pSer^144^ showed a median survival of 0.6 years whereas this value was 3.35 years for those with lower PAK1 pSer^144^. These data indicate that, on average, patients with low PAK1 activity lived 5 times longer than those with high activation of this kinase and give weight to the notion that PAK1 is a suitable target for the treatment of AML cases that respond poorly to current treatments.

### PF-3758309 was the most effective PAK inhibitor in reducing cell proliferation and inducing apoptosis in AML cell lines

Initially, we tested the sensitivity of P31/Fuj and MV4-11 cells to three PAK inhibitors that target PAK1. In MV4-11 cells, 0.1 µM PF-3758309, 1 µM FRAX-486 and 20 µM IPA-3 blocked proliferation and induced apoptosis, while in P31/Fuj, 1 µM PF-3758309, 10 µM FRAX-486 and 20 µM IPA-3 blocked proliferation and 10 µM PF-3758309 and FRAX-486 and 20 µM IPA-3 induced apoptosis. (Fig. 1C and D). These data show that MV4-11 cells were more sensitive than P31/Fuj cells to the reduction of cell proliferation and the increase of apoptosis induced by PF-3758309 and FRAX-486 (Fig. 1). Relevantly, PF-3758309 inhibited cell proliferation and induced cell death more efficiently than FRAX-486 and IPA-3 in both cell lines (Fig. 1C and D). Therefore, we decided to focus our study on characterizing how AML cells response to PF-3758309 at the molecular level.

### Identification of kinases and other proteins targeted by PF-3758309 in AML cell lines

**Fig. 2:**
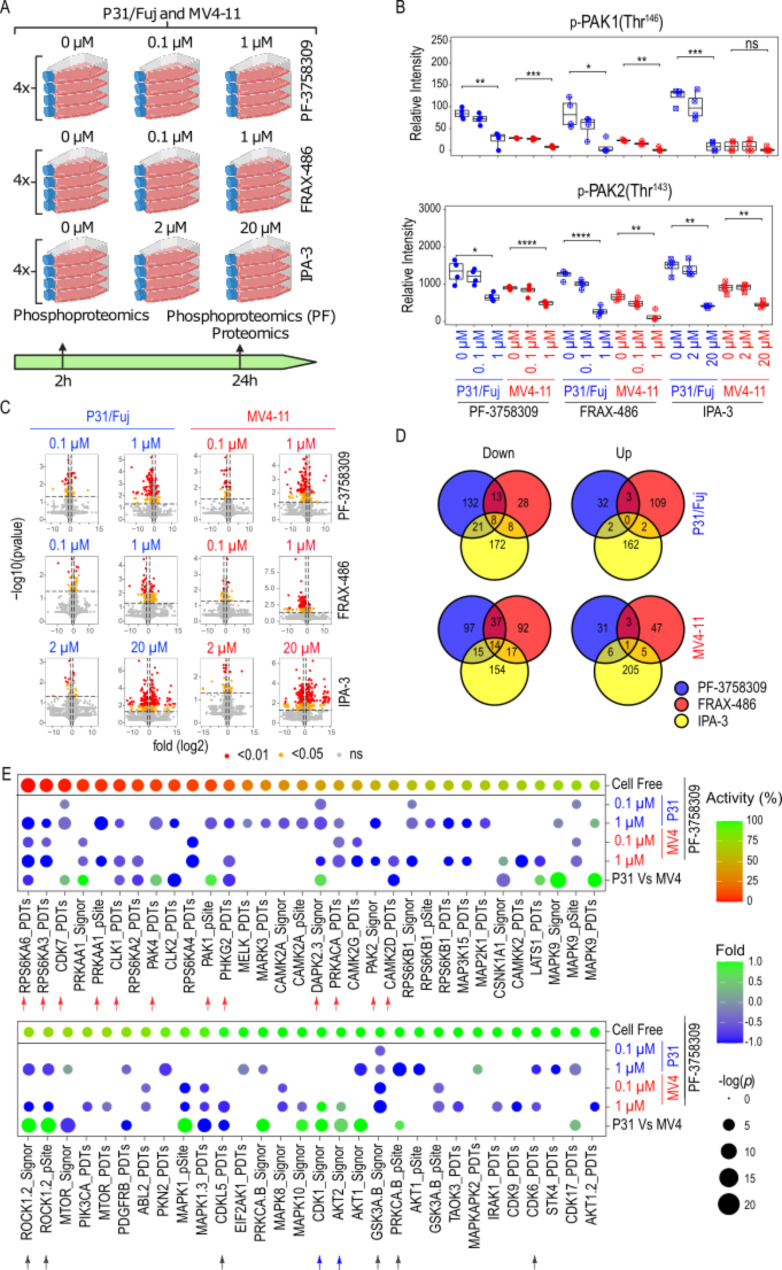
Identification of kinases targeted by PF-3758903 in AML cell lines after 2 h treatment. **A**. Scheme of the phosphoproteomics and proteomics experiments detailing replicates, cell lines, compounds, concentrations and exposure times for the analysed conditions. **B**. Inhibition of auto-phosphorylation sites in PAKs after 2 h treatment with PF-3758309, FRAX-486 and IPA-3 in P31/Fuj and MV4-11 cells. **C**. Volcano plots indicating phosphopeptides affected by 2 h treatment with PF-3758309, FRAX-486 and IPA-3 in P31/Fuj and MV4-11 cells. **D**. Number of phosphopeptides commonly up or down regulated across PAK inhibitors after 2 h treatment in P31/Fuj and MV4-11 cells. **E**. Estimation of kinase activity using KSEA after 2 h treatment with the indicated concentrations of PF-3758309. Boxplots indicate median, 1st and 3rd quartiles. Whiskers extends from the hinge to the largest and lowest value no further than 1.5 times the distance between the 1st and 3rd quartiles. In **B**, Data points represent individual replicates. In **C**, data points represent individual phosphopeptides. Statistical significance was calculated using unpaired two-sided Student’s t-test (*n* = 4 independent experiments). **** *p* ≤ 0.0001, *** *p* ≤ 0.001, ** *p* ≤ 0.01 and * *p* ≤ 0.05

To identify kinases and other proteins that could explain the impact of PAK inhibitors in the proliferation and apoptosis of these AML cell lines, we profiled these compounds using proteomic and phosphoproteomic approaches. We treated P31/Fuj and MV4-11 cells with the indicated concentrations of PF-3758309, FRAX-486 and IPA-3 (Fig. 2A) that were selected based on their functional effects (Fig. 1). We collected extracts after 2 h treatment for phosphoproteomics analysis and after 24 h for phosphoproteomics (PF-3758309 treatments only) and proteomics in biological quadruplicate (Fig. 2A).

Quality control analysis showed that average peptide and protein intensities in all samples centred close to 0 in all 3 experiments (Supplementary Fig. 1A-C). Phosphoproteomic studies identified 3,788 and 2,369 phosphopeptides in cells exposed to 2 and 24 h treatments, respectively, while proteomics analysis identified 1,596 proteins (Supplementary Fig. 1D; Additional File 1).

As positive controls for the experiments, we observed that treatment for 2 h with 1µM PF-3758309 and FRAX-486 reduced the autophosphorylation of PAK1 at Thr^146^ and PAK2 at Thr^143^ in P31/Fuj and MV4-11 cells, while 2 h treatment with 20 µM IPA-3 also reduced the autophosphorylation of PAK2 in both cell lines and of PAK1 in P31/Fuj (Fig. 2B). This indicates that our treatments with these compounds were effective in inhibit PAK isoforms in both cell lines.

We observed a dose dependent increase in the number of protein residues that significantly increased or decreased their phosphorylation after 2 h treatment with all three compounds (Fig. 2C). Although all 3 inhibitors targeted PAKs (Fig. 2B), we found that that the phosphorylation of only 8 and 14 peptides was commonly reduced by PF-3758309, FRAX-486 and IPA-3 in P31/Fuj and MV4-11 cells, respectively (Fig. 2D). Similarly, only the phosphorylation of 1 peptide was increased by all inhibitors in P31/Fuj cells (Fig. 2D). PAK isoform specificity or different off-target effects across compounds could explain the lack of commonly targeted peptides.

To determine the kinases targeted by PF-3758309 treatment in P31/Fuj and MV4-11 cells in vivo, we applied KSEA [11, 18] to our phosphoproteomics data using the PDTs, pSite and Signor databases (Fig. 2E, rows 2 to 5). To infer kinase activities directly inhibited by PF-3758309 relative to those that may be downstream of the primary targets, we also considered the inhibitory effect of PF-3758309 in vitro in a cell free kinase activity assays [18] (Fig. 2E, top row in the dot plot). Finally, we calculated differences in estimated kinase activity between P31/Fuj and MV4-11 to determine whether differences in basal kinase activity could explain cell specific inhibitory effects of PF-3758309 (Fig. 2E, row 6).

Exposure to 1 µM PF-3758309 inhibited the activity of PAK2 (Signor) in both cell lines and PAK4 (PDTs) and PAK1(pSite) in P31/Fuj cells, which presented higher activities for these kinases (Fig. 2E). Cell free assays also showed a high inhibitory activity of PF-3758309 on PAK4, PAK1 and PAK2 (Fig. 2E). FRAX-486 inhibited only PAK2 in MV4-11 and IPA-3 inhibited PAK1and PAK2 only in P31/Fuj cells. In addition, 1 µM PF-3758309 inhibited more efficiently PAK4 activity than 20µM IPA-3 in P31/Fuj cells (Supplementary Fig. 2). These results are consistent with our previous data indicating that PF-3758309 is the most potent PAK inhibitor, with FRAX-486 and IPA-3 targeting mainly Group I PAKs and PF-3758309 targeting both Classes.

PF-3758309 also inhibited RPS6KA6 (PDTs), RPS6KA3 (PDTs), PRKAA1/AMPK (pSite), CLK1 (PDTs), PHKG2 (PDTs), DAPK (Signor) and PRKA (PDTs) in both cell lines, together with CDK7 (PDTs) in P31/Fuj and CAMK2D (PDTs) in MV4-11 that, respectively, over activate these kinases (Fig. 2E, red arrows). Cell free assays suggest that these kinase activities are directly inhibited by PF-3758309 (Fig. 2E, row 1). PF-3758309 inhibited ROCK1/2 (Signor and pSite), GSK3A/B (Signor) and CDK6 (PDTs) in both cell lines together with the overactive PKCA/B (pSite) in P31/Fuj and CDK5L (PDTs) in MV4-11 (Fig. 2E, black arrows), but the lack of effect of PF-3758309 on these kinases in cell free assays suggest that these kinase activities are indirectly inhibited by PF-3758309 (Fig. 2E, top row). In addition, PF-3758309 increased the activity of mTOR (PDTs) and MAPKAP2 (PDTs) in P31/Fuj and CDK1 (Signor) and AKT2 (Signor) in MV4-11 (Fig. 2E, blue arrows).

Together, these results indicate that in AML cell lines, PF-3758309 inhibited the kinases PAK, RPS6KAs, AMPK, PKA, CLKs and PHKG2 directly, whereas ROCKs, PKC and CDK5L were reduced as a downstream knock-on effect.

### Prolonged treatment with PF-3758309 impact kinase activities that are specifically elevated in AML cell models

**Fig. 3:**
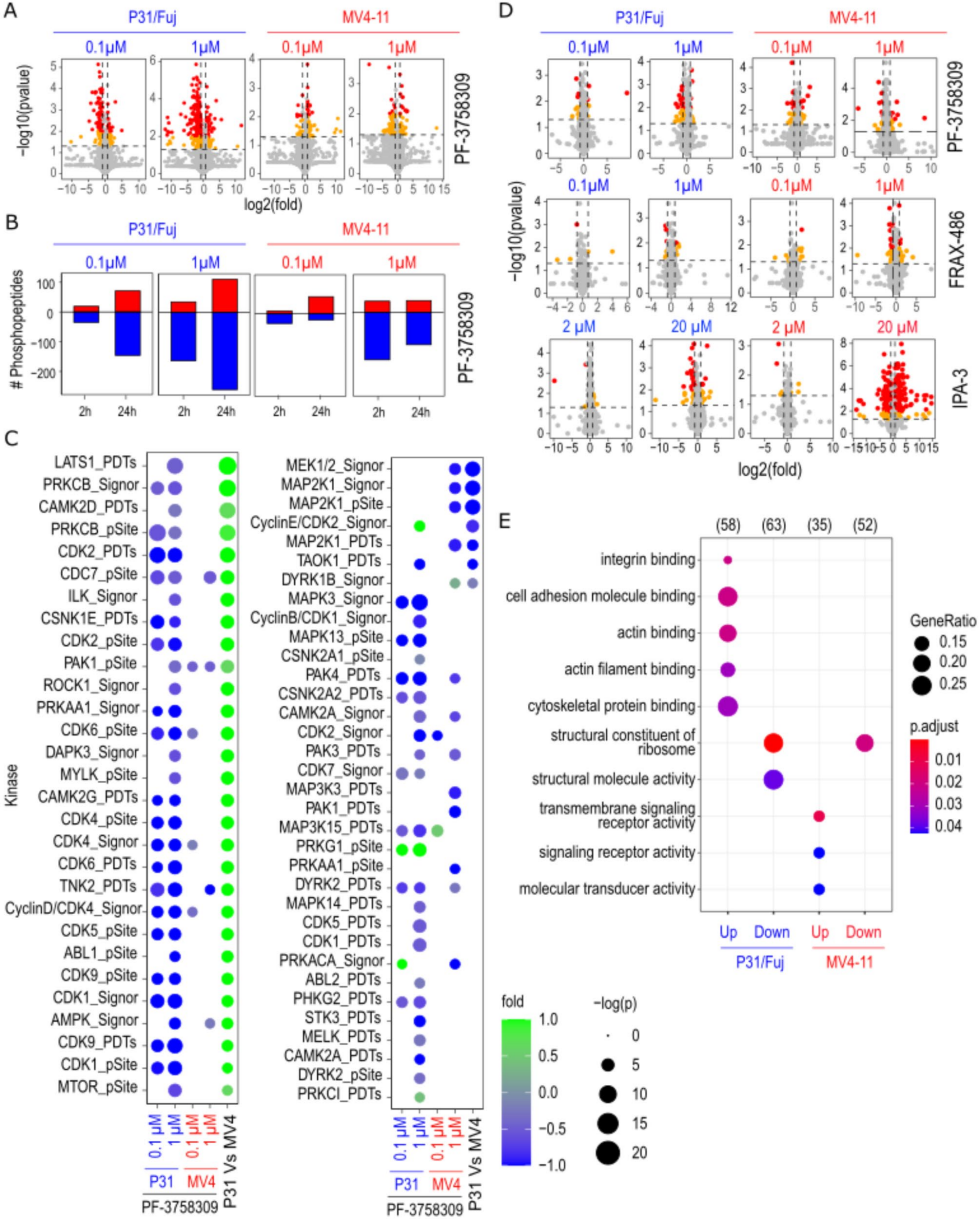
Identification of kinases and gene ontologies targeted by PF-3758309 after 24 h treatment. **A**. Volcano plots indicating the phosphopeptides affected by 24 h treatment with the PAK inhibitors PF-3858309 in P31/Fuj and MV4-11 cells. **B**. Bar plots showing the number phosphopeptides affected by 2 and 24 h treatment with the PAK inhibitors PF-3858309 in P31/Fuj and MV4-11 cells. **C**. Estimation of kinase activity using KSEA after 24 h treatment with 0.1 and 1 µM of PF-3758309. **D**. Volcano plot showing the proteins up or down regulated after 24 h treatment with the indicated concentrations of PF-3758309, FRAX-486 and IPA-3 in P31/Fuj and MV4-11 cells. **E**. Gene ontologies enrich in the set of proteins up or down regulated after 24 h treatment with PF-3758309 in P31/Fuj and MV4-11 cells. In **A** and **D**, data points represent individual phosphopeptides and proteins, respectively. For **A**-**D**, Statistical significance was calculated using unpaired two-sided Student’s t-test (*n* = 4 independent experiments). For **E**, statistical significance was calculated using a modified Fisherman’s test

To investigate how cells respond to prolonged PAKi perturbation at the protein phosphorylation level, we studied the effect of PF-3758309 on kinase activity 24 h after treatment. PF-3758309 modified the phosphorylation of multiple peptides in a dose dependent manner in P31/Fuj and MV4-11 cells (Fig. 3A), and, as expected, the reduction in protein phosphorylation after 1 µM treatment was more pronounced after 24 h than after 2 h in both cell lines (Fig. 3B).

KSEA showed that 1 µM PF-3758309 still reduced the activity of PAK1 (pSite), PAK4 (PDTs), AMPK (Signor) and CAMK2A (Signor) 24 h after treatment in both cell lines. Conversely, no effect on any RPS6KA activity was detected after 24 h treatment with PF-3758309 in any cell line (Fig. 3C). In addition, PAK3 (PDTs), CDC7 (PDTs), DYRK2 (PDTs) and TNK2 (PDTs) activities were reduced 24 h after treatment with 1 µM of PF-3758309 in both cell lines (Fig. 3C). Treatment with 1 µM PF-3758309 inhibited multiple kinase activities that were overactive in the respective cell line relative to the other model. For example, in P31/Fuj cells, this compound reduced the activity of ROCK, PKCs and multiple CDKs, which were more active in this cell line than in MV4-11, while MEK1 and TAOK1 activities were found to be increased in MV4-11 and inhibited by the compound (Fig. 3C). In summary, these data show that activities of PAK, AMPK and CAMK2 that were inhibited after 2 h treatment with PF-3758309 were also inhibited after 24 h treatment. Furthermore, PF-3758309 inhibited a markedly different pattern of kinase activities across P31/Fuj and MV4-11 cells after 24 h treatment, and this inhibition pattern heavily depended on the basal kinase activities in each cell line.

### PF-3758309 impact the phosphorylation of proteins involved in cell signalling and epigenetic regulation

To investigate how PF-3758309 may affect protein expression, we next investigated how this compound impact the phosphorylation of factors involved in transcription, translation and protein degradation. We found that, in P31/Fuj and MV4-11 cells, treatment with PF-3758309, FRAX-486 and IPA-3 altered the levels of multiple proteins in a dose dependent manner (Fig. 3D). Gene ontology (GO) analysis showed reduced the levels of structural components of the ribosomes in both cell lines (Fig. 3E), including multiple proteins of the 60 S and 40 S ribosomal subunits as well as several translation initiation and elongation factors (Supplementary Fig. 2). Additionally, PF-3758309 increased the expression of proteins involved in cell adhesion and cytoskeletal biology in P31/Fuj cells and the expression of proteins linked to signal transduction in MV4-11 cells (Fig. 3E). In summary, our results indicate that PF-3758309 affects protein expression on ontologies associated to a reduction of protein translation in both cell lines, and an increase in ontologies associated to cell adhesion in P31/Fuj and signal transduction in MV4-11 cells.

**Fig. 4:**
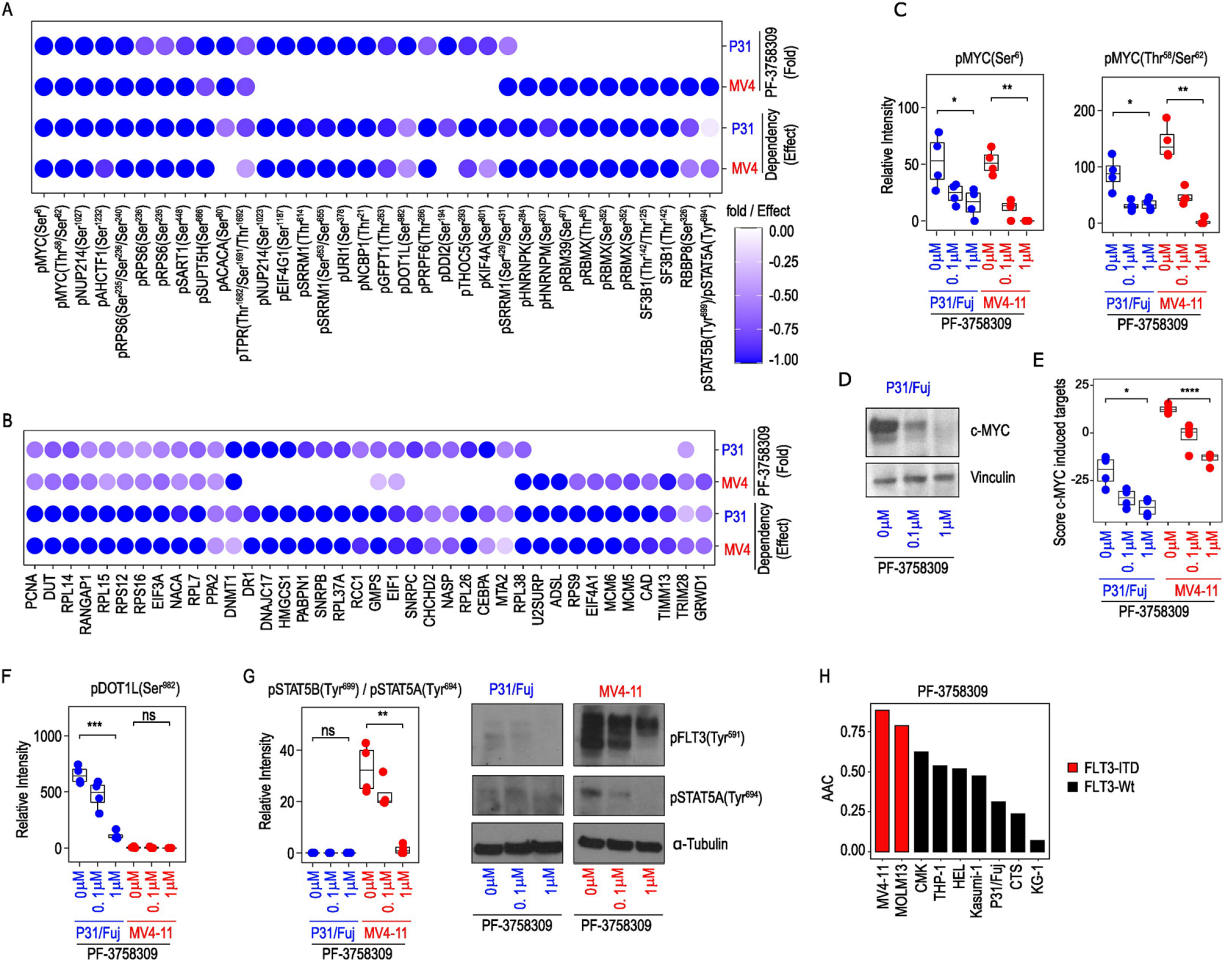
PF-3758309 targeted c-MYC, DOT1L and FLT3-ITD. **A**. PF-3758309 reduced the phosphorylation of proteins relevant for the proliferation of AML cells. **B**. PF-3758309 reduced the and expression of proteins relevant for the proliferation of AML cells. **C**. Treatment of P31/Fuj and MV4-11 with PF-3758309 for 2 h reduced the phosphorylation of c-MYC. **D**. Treatment of P31/Fuj with PF-3758309 for 24 h reduced the expression of c-MYC. **E**. Treatment of P31/Fuj and MV4-11 cells with PF-3758309 for 24 h reduced the activity of c-MYC. **F**. Treatment of P31/Fuj cells with PF-3758309 for 24 h reduced the phosphorylation of DOT1L at Ser^962^. **G**. Treatment of MV4-11 cells with PF-3758309 for 24 h reduced the phosphorylation of STAT5A at Tyr^694^ and FLT3 at Tyr^591^. **H**. AML cell lines positive for FLT3-ITD mutations are more sensitive to PF-3758309. Boxplots indicate median, 1st and 3rd quartiles. Whiskers extends from the hinge to the largest and lowest value no further than 1.5 times the distance between the 1st and 3rd quartiles. Data points represent individual replicates. Statistical significance was calculated using unpaired two-sided Student’s t-test (*n* = 4 independent experiments). **** *p* ≤ 0.0001, *** *p* ≤ 0.001, ** *p* ≤ 0.01 and * *p* ≤ 0.05

To identify PF-3758309 targeted proteins that could play key roles in the biology of AML cells, we filtered the phosphoproteomics data by integrating changes in protein phosphorylation and expression with gene dependency data from the DepMap portal focusing on the RNAi Achilles screening covering 501 cell lines and 17,098 genetic knock downs (Fig. 4A and B). The reasoning for taking this approach is that not all proteins that reduced their phosphorylation after PAKi treatment may be involved in the antiproliferative effects of the compound. However, those that are involved in regulating survival downstream of PAK should also produce an impact on cell viability, measured as a dependency score, when targeted genetically in the specific cell line. In P31/Fuj and MV4-11 cells, PF-3758309 reduced the phosphorylation of c-MYC, NUP214, AHCTF1, RPS6, SART1, SUPTH5 and the expression of PCNA, DUT, RANGAP, EIF3A, NACA and the ribosomal proteins L7, L14, L15, S12 and S16. DepMap analysis showed that these proteins are needed for the proliferation of both cell lines (Fig. 4A and B). PF-3758309 also reduced, in a cell model-specific manner, the phosphorylation and expression of proteins that are important for the proliferation of at least the respective model. These include NCBP1 and DOT1L phosphorylation and DR1 and DNAJC17 expression in P31/Fuj, and RBMX and STAT5B phosphorylation and MCM5 and MCM6 expression of in MV4-11 cells (Fig. 4A and B). These data confirm that PF-3758309 has a pleotropic a mode of action in AML cells and identify proteins inhibited by the compound in cell-specific manner with roles in regulating cell viability.

### PAK inhibitors reduce c-MYC phosphorylation, expression and transcriptional activity

c-MYC is an important but undruggable oncogene with numerous roles in cancer progression [24]. Interestingly, we found that treatment with PF-3758309 for 2 h reduced the phosphorylation of c-MYC at Ser^6^, Thr^58^ and Ser^62^ in P31/Fuj and MV4-11 cells (Fig. 4C, left panel). Of note, phosphorylation of c-MYC at Ser^62^ is key for the functions of this transcription factor as it promotes its stability and transcriptional activity [24]. Consistently, we found that treatment with PF-3758309 for 72 h reduced the protein levels of c-MYC in P31/Fuj cells in a dose dependent manner (Fig. 4D). Similarly, estimation of transcription factor activity, using protein expression of c-MYC regulated genes (Supplementary Fig. 3), showed that PF-3758309 reduced c-MYC transcriptional activity after 24 h treatment (Fig. 4E). FRAX-486 and IPA-3 also reduced the phosphorylation of c-MYC in MV4-11 cells and tended to reduce it in P31/Fuj cells (Supplementary Fig. 4A). FRAX-486 only reduced the transcriptional activity of c-MYC in MV4-11 cells while IPA-3 did not produce any significant change in the activity of this transcription factor (Supplementary Fig. 3). PF-3758309 almost completely abrogated c-MYC expression as early as 2 h after treatment, while inducing a small increase in CleavedPARP at this time point (Supplementary Fig. 4B). However, induction of apoptosis by PF-3758309 was minimal relative to the extent by which navitoclax triggers this process in this model (Supplementary Fig. 4B). Since the reduction of c-MYC levels induced by PF-3758309 occurred before triggering full scale cell death, these data suggest that the effects of PF-3758309 on c-MYC are not a consequence of the apoptotic process caused by the compound (Supplementary Fig. 4B and C). These data indicate that targeting PAK with small molecule inhibitors provides a means to reduce the transcriptional activity of c-MYC, an important but otherwise difficult to drug oncogene.

### Mechanistic heterogeneity of PF-3758309 mode of action

To explore the mechanistic heterogeneity by which targeting PAK impacts the viability of cancer cells with different genetic backgrounds, we investigated the effect of PF-3758309 in key proteins specifically deregulated in each cell line. P31/Fuj cells present a PICALM-MLLT10 translocation that leads to the deregulated activity of the MLLT10 partner DOT1L [25, 26]. We found that P31/Fuj cells hyper-phosphorylated (relative to MV4-11) DOT1L at Ser^982^ and treatment with PF-3758309 reduced this phosphorylation (Fig. 4F). FRAX-486 but not IPA-3 also reduced the phosphorylation of DOT1L at Ser^982^ in P31/Fuj cells (Supplementary Fig. 4D). Conversely, MV4-11 cells present an ITD mutation in the receptor tyrosine kinase FLT3 that produce a hyper-activated version of the enzyme [27]. FLT3-ITD phosphorylates STAT5B at Tyr^699^ and, of note, PF-3758309 reduced this phosphorylation (Fig. 4G, left panel). Consistent with the mass spectrometry data, Western blot analysis proved that PF-3758309 reduced the phosphorylation of STAT5B at Tyr^699^ and FLT3 at Tyr^591^ in MV4-11 cells (Fig. 4G, right panels).

To functionally evaluate the effect of PF-3758309 over the activity of FLT3, we treated a panel of 9 AML cell lines with increasing concentrations of PF-3758309 (Supplementary Fig. 4G) and estimated the area above the curve (AAC) of a cell viability assay. The FLT3-ITD positive cell lines MV4-11 and MOLM-13 [28] were the most sensitive cell lines to the kinase inhibitor (Fig. 4H and Supplementary Fig. 4G). In MV4-11 cells, FRAX-486 also reduced the phosphorylation of STAT5B at Tyr^699^ and IPA-3 tended to reduce it too (Supplementary Fig. 4E). Western blot confirmed that both inhibitors reduced the phosphorylation of FLT3 at Tyr^591^ and STAT5B at Tyr^699^ (Supplementary Fig. 4F).

In summary, these data demonstrate that PF-3758309 inhibits oncoproteins frequently deregulated in AML, like c-MYC, and proteins directly deregulated by specific genomic alterations, like DOT1L, in cells with PCAM-MLLT10 rearrangements and FLT3 in cells with FLT3-ITD mutations.

### Cellular functions targeted by PF-3758309

AML is characterized by a dysregulation of the balance that exists between cell differentiation and proliferation/cell cycle progression. Given that PF-3758309 inhibits the phosphorylation of epigenetic enzymes and transcription factors, such as DOT1L and c-MYC, involved in these processes, we asked whether these molecular alterations were producing functional changes in cells treated with the PAKi. Thus, in addition to proliferation and apoptosis (Fig. 1), we examined to what extent PF-3758309 modulates cell cycle progression and differentiation in AML cells.

**Fig. 5:**
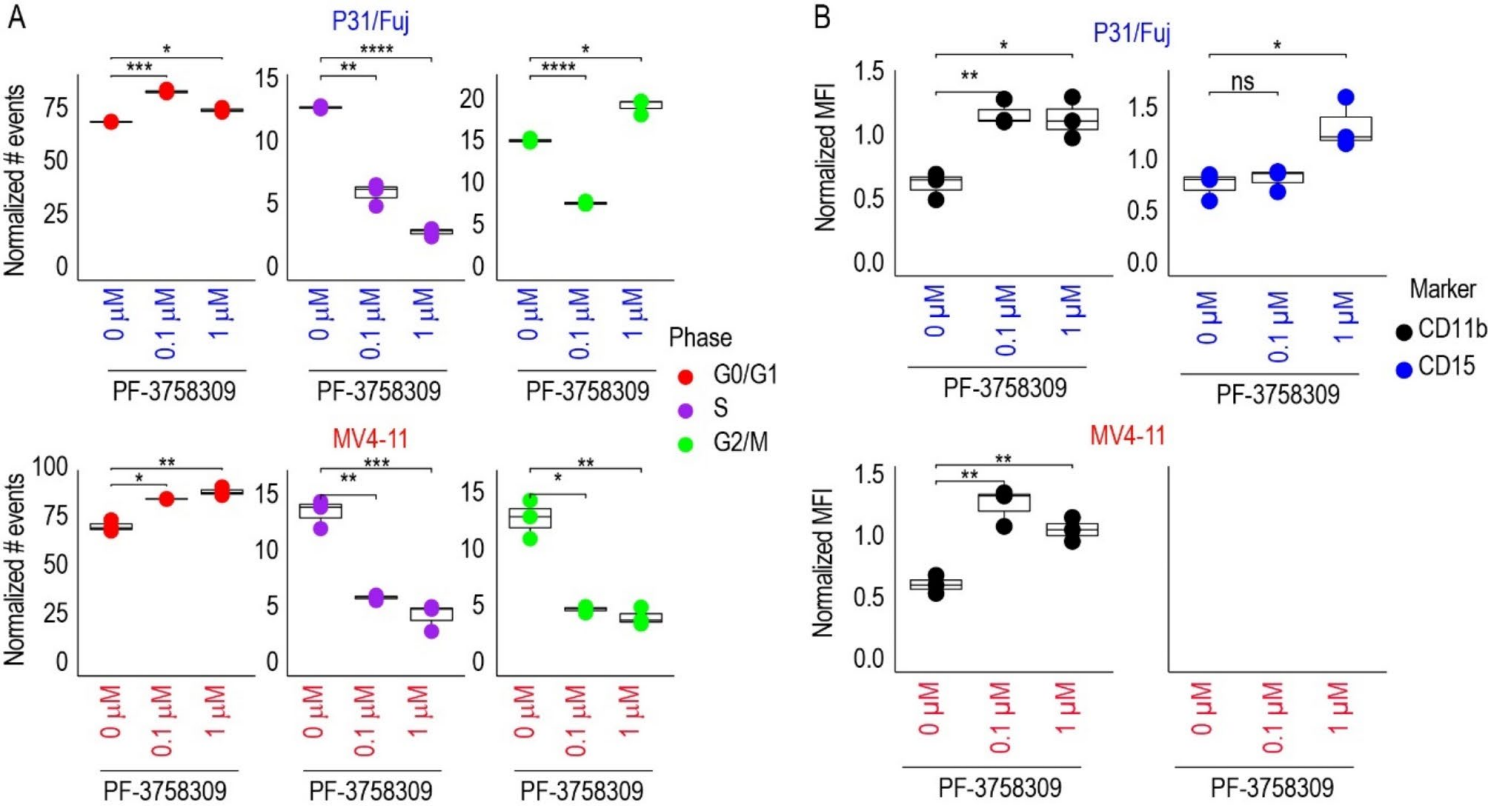
PF-3758309 blocked cell cycle progression and promoted differentiation in AML cell lines. **A**. Treatment with PF-3758309 for 24 h blocked the cell cycle at G_0_/G_1_ or G_0_/G_1_ and G_2_/M phases in P31/Fuj cells and at G_0_/G_1_ in MV4-11 cells. **B**. Treatment with PF-3758309 for 72 h increased the expression of the differentiation markers CD11b in P31/Fuj and MV4-11 cells and CD15 in P31/Fuj cells. Boxplots indicate median, 1st and 3rd quartiles of normalise fluorescence intensities. Whiskers extends from the hinge to the largest and lowest value no further than 1.5 times the distance between the 1st and 3rd quartiles. Data points represent individual replicates. Statistical significance was calculated using unpaired two-sided Student’s t-test (*n* = 3 independent experiments). **** *p* ≤ 0.0001, *** *p* ≤ 0.001, ** *p* ≤ 0.01 and * *p* ≤ 0.05

Treatment with 0.1 and 1 µM PF-3758309 reduced the number of cells at the S phase of the cell cycle in P31/Fuj and MV4-11 cells (Fig. 5A), a finding consistent with a reduction in the activity of the transcription factor E2F1 in both cell lines (Supplementary Fig. 3). Treatment of MV4-11 with 0.1 or 1 µM and P31/Fuj with 0.1 µM reduced the G_2_/M faction and increased the G_0_/G_1_ indicating that PF-3758309 blocked or delayed the advance of the cell cycle in G_0_/G_1_ phases (Fig. 5A). Treatment of P31/Fuj cells with 1 µM of the inhibitor increased the fraction of cells in G_0_/G_1_ and G_2_/M phases suggesting an additional effect of this concentration in the G2/M phase (Fig. 5A).

As for its effects on cell differentiation, we found that PF-3758309 increased the surface expression of myeloid markers CD11b in both cell lines and CD15 in P31/Fuj (Fig. 5B), which is in line with an increase in the activity of the transcription factors CEBPA and CEBPB in P31/Fuj cells and SPI1 (also known as PU.1) in both cell lines after PF-3758309 treatment (Supplementary Fig. 3). In summary, our functional data indicate that PF-3758309 targeted the cell cycle progression mainly at G_0_/G_1_ but also at G_2_/M phases and induced differentiation in AML cell lines.

### Identification of kinases and other proteins targeted by PF-3758309 in AML primary cells

Kinase signalling networks can differ between AML cell lines and primary blast. Therefore, we studied how 2 h ex vivo treatment with 0.1 or 1 µM PF-3758309 modulated protein phosphorylation and kinase activity of mononuclear cells extracted from the bone marrow or peripheral blood of 8 AML patients (Additional File 2).

**Fig. 6:**
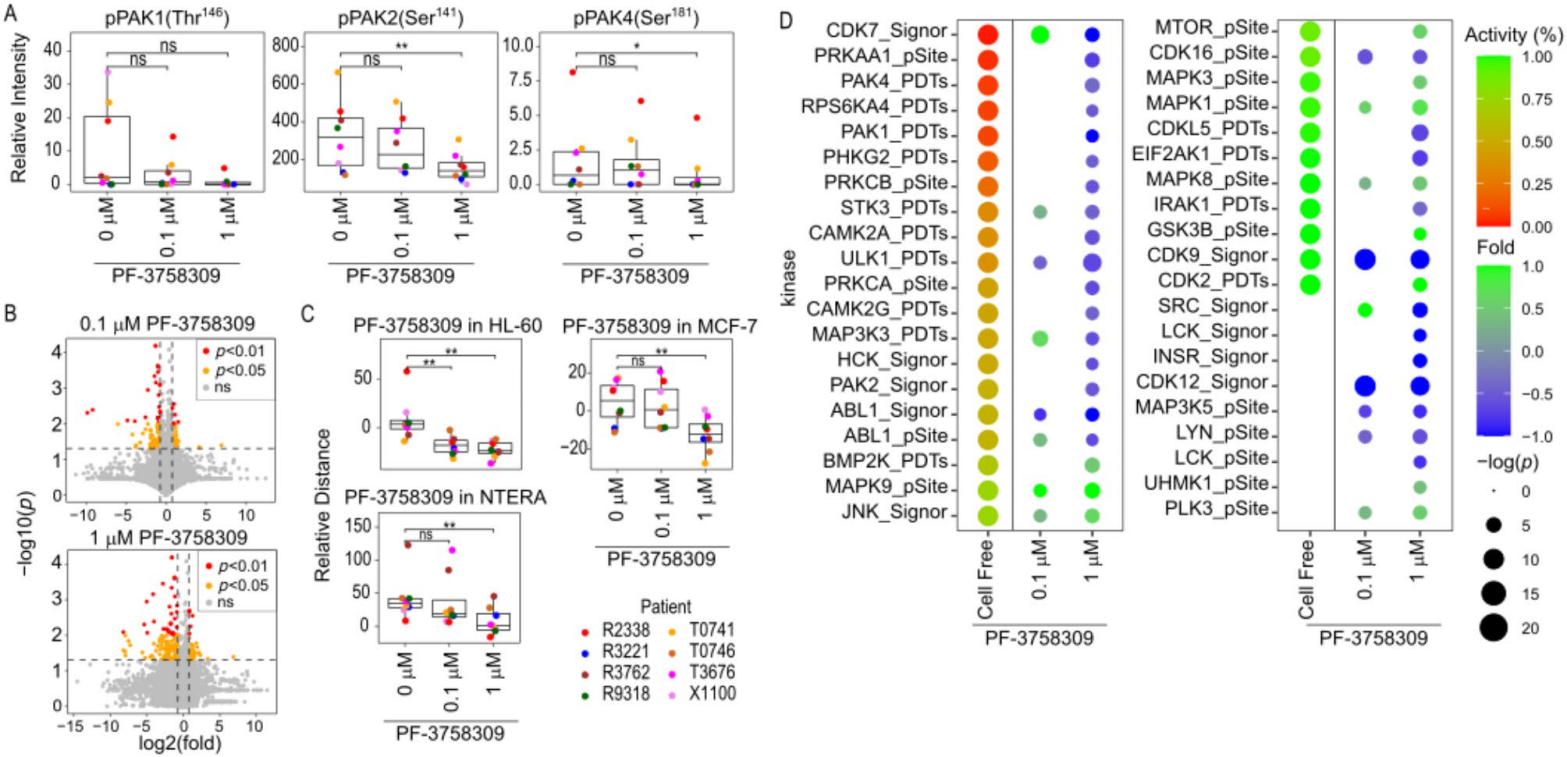
PF-3758309 targeted PAKs and other kinases in AML primary cells. **A**. PF-3758309 reduced the auto-phosphorylation of PAK1, PAK2 and PAK4 in AML primary blast. **B**. Volcano plot showing phosphopeptides regulated by 2 h treatment with PF-3758309 in AML primary cells. **C**. Treatment with PF-3758309 reduced in AML primary cells the phosphorylation of the sets of peptides inhibited by PF-3758309 in HL-60, MCF-7 and NTERA-2 cells. **D**. Estimation of kinase activity using KSEA after 2 h treatment with 0.1 and 1 µM of PF-3758309 in AML primary cells, activity indicates the ability of PF-3758309 to inhibit the indicated kinase in cell-free assays. In **A**, **C** and **D**, data points represent individual patient observations. Boxplots indicate median, 1st and 3rd quartiles. Whiskers extends from the hinge to the largest and lowest value no further than 1.5 times the distance between the 1st and 3rd quartiles. In **B**, data points represent individual phosphopeptides. Statistical significance was calculated using unpaired two-sided Student’s t-test (*n* = 4 independent experiments). **** *p* ≤ 0.0001, *** *p* ≤ 0.001, ** *p* ≤ 0.01 and * *p* ≤ 0.05

Quality control assessment showed that the average of the normalized peptide intensities across all samples centred close to 0 (Supplementary Fig. 5A). Reassuringly, PF-3758309 treatment reduced the autophosphorylation of PAK2 at Ser^141^ and PAK4 at Ser^181^ (Fig. 6A). PAK1 autophosphorylation at Thr^146^ and the FLT3 associated phosphorylation of STAT5B at Tyr^699^ were also reduced in patients that presented detectable levels of phosphorylation at these sites (Fig. 6A; Supplementary Fig. 5B). As expected, PF-3758309 reduced peptide phosphorylation in a dose dependent manner (Fig. 6B). As an additional metric of data quality, compound target activity marker (CTAM) analysis, an algorithm that mines phosphorylation sites known to be inhibited by particular compounds [18, 20], showed that PF-3758309 treatment reduced the phosphorylation of the sites that were previously found to be reduced by PF-3758309 in other cell lines (HL-60, MCF-7 and NTERA-2 cells, Fig. 6C), highlighting the consistency of our phosphoproteomic assay.

KSEA analysis showed that, similar to the results in cell lines, PF-3758309 directly reduced the activity of PAK1 (PDTs), PAK2 (Signor) and PAK4 (PDTs) in AML primary cells and indirectly targeted the kinase CDKL5 that is also involved in cytoskeletal reorganization [29]. In addition, PF-3758309 inhibited the activity of PRKAA1/AMPK and its related kinases ULK1 and CAMK2, RPS6KA4 and CDKs that target the RNA polymerase 2 (CDK7, CDK12 and CDK16) (Fig. 6D). Indeed, PF-3758309 strongly reduced the phosphorylation of the RNA polymerase 2 at the C-terminal domain (Supplementary Fig. 5C). Other kinases inhibited by PF-3758309 included PKC isoforms, multiple tyrosine kinases (ABL, HCK, INSR, LCK, LYN and SRC), STK3, PHKG2 and several kinases involved in stress response (IRAK, MAP3K3, MAP3K5 and EIF2AK) (Fig. 6D). Conversely, PF-3758309 increased the activity of JNKs (MAPK8 and MAPK9), PLK3, ERKs (MAPK1 and MAPK3) and mTOR (Fig. 6D) that can be involved in stress response and drug resistance, together with GSK3B, CDK2, UHMK1 and BMP2K (Fig. 6D).

In line with these results, CTAM analysis showed that PF-3758309 reduced the phosphorylation of peptides targeted by the CAMK2 inhibitor KN-93, and the PKC and PLC (activator of PKC) inhibitors GF-109203X, Go-6983 and U-73122 (Supplementary Fig. 5D). CTAM analysis also showed that PF-3758309 increased the phosphorylation of peptides targeted by the ERK and MEK (activator of ERK) inhibitors GDC-0994 and Trametinib, respectively (Supplementary Fig. 5D).

In summary, our data showed that the targets of PF-3758309 in AML primary cells and cell lines were similar. Furthermore, in addition to PAKs, PF-3758309 inhibited the activity of AMPK, CAMK2, CDKs, PKC and multiple tyrosine kinases, while increasing the activity of MTOR and MAPKs in AML primary blast, suggesting that potential mechanisms of acquired resistance involving these pro-survival and mitotic kinases may operate in primary cells.

### Kinase activities and phosphorylation sites inhibited by PF-3758309 are associated with responses to the compound in primary AML cells

Although PF-3758309 blocked cell proliferation and the induced differentiation and apoptosis, these effects were not homogeneous across AML cell lines and patient samples. Therefore, the implementation of a successful targeted therapy in AML using PAK inhibitors would require the identification of patients more likely to present leukaemic cells sensitive to treatment with a PAKi.

To investigate determinants of sensitivity, we used a previously published phosphoproteomics and proteomics datasets on primary blast from 36 AML patients with known ex vivo responses to 1 µM treatment with PF-3758309 [14]. Initially, we used a knowledge based approached to regress sensitivity to PF-3758309 against the expression, activity and phosphorylation of the kinases targeted by the compound in cell line models and primary AML cases.

**Fig. 7:**
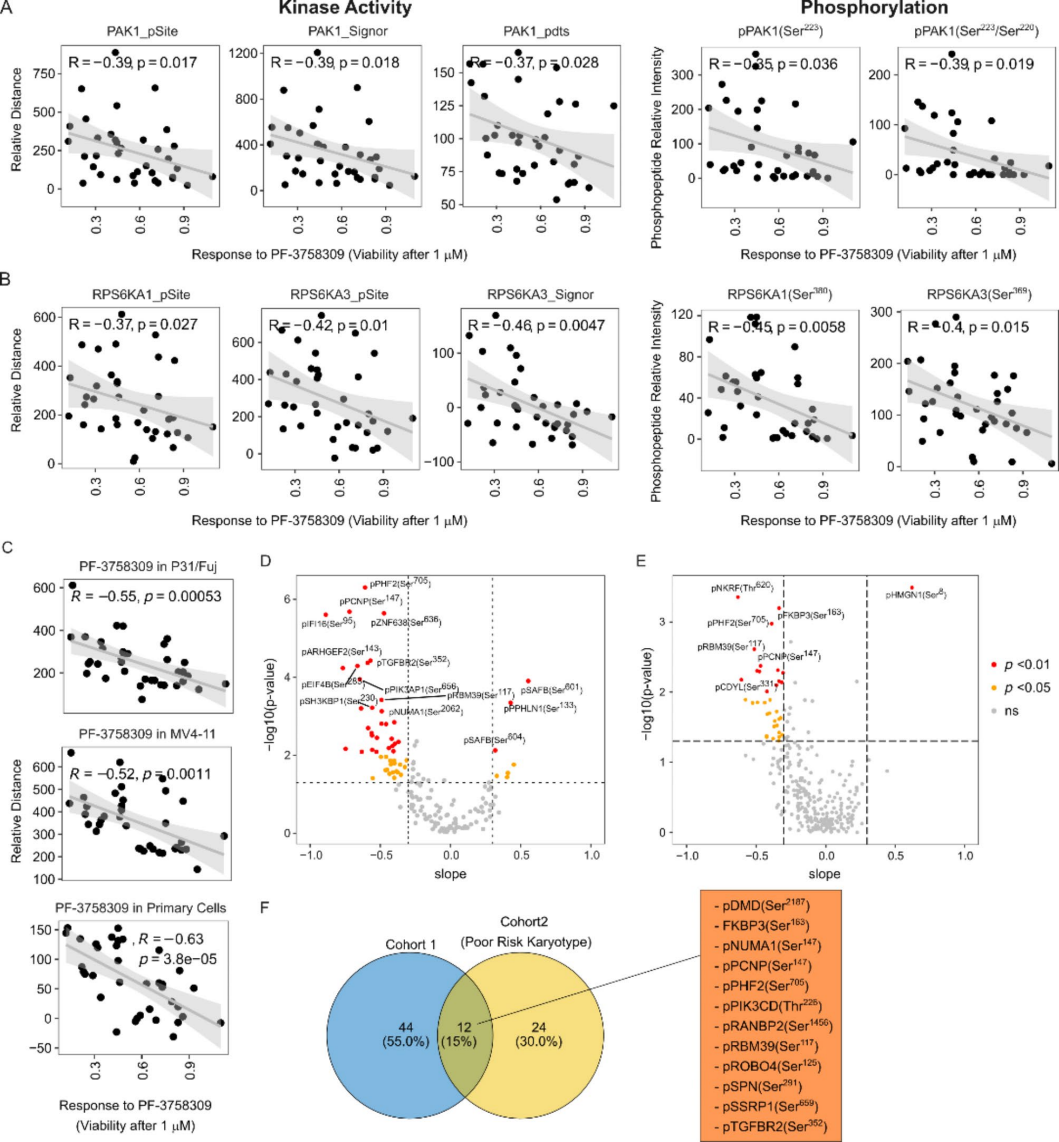
PF-3758309 sensitivity correlates with PAK1 and RPS6KA activity and phosphorylations targeted by PF-3758309. **A**. Correlation between PF-3758309 sensitivity and PAK1 activity and phosphorylation in primary AML cells. **B**. Correlation between PF-3758309 sensitivity and RPS6KAs activity and phosphorylation in primary AML cells. **C**. Correlation, in primary AML cells, between PF-3758309 sensitivity and CTAM scores for phosphorylations targeted by PF-3758309. **D**. Correlation in primary AML cells [14] between sensitivity to PF-3758309 and peptide intensity for phosphopeptides targeted by PF-3758309 in primary cells. **E**. Correlation in an independent cohort of primary AML cells [22] between sensitivity to PF-3758309 and peptide intensity for phosphopeptides targeted by PF-3758309 in primary cells. **F**. Overlap between phosphopeptides significant in **D** and **E**. Correlation was determined using Spearman correlation values

Linear regression revealed no correlation between protein expression of PAK isoforms and PF-3758309 sensitivity (Supplementary Fig. 6). In contrast, high PAK1 activity was significantly (*p* < 0.02) increased in cases sensitive to the compound (Fig. 7A and Supplementary Fig. 6A). Similarly, although the phosphorylation of PAK1 at Ser^144^ did not correlate with compound response, phospho-Ser^223^, a site that is also related to PAK1 activity [30], was associated with higher sensitivity to PF-3758309 (Fig. 7A and Supplementary Fig. 6A). We also observed that the phosphorylation of targets downstream of PAK2 and PAK4 were, respectively, linked to resistant and sensitivity to PF-3758309 and the uncharacterized PAK2 phosphorylation sites at Ser^58^ and Ser^64^ also correlated with sensitivity to the compound (Supplementary Fig. 6B and C).

As for ribosomal protein kinases, which are also targets of PF-3758309, we found that RPS6KA1 and RPS6KA3 activities (pSite or Signor), but not RPS6KB (Supplementary Fig. 7A), and activatory phosphorylation sites (RPS6KA1 at Ser^380^ and RPS6KA3 at Ser^369^) [31, 32] were significantly elevated in primary cells sensitive to the compound, while the phosphorylation of sites downstream of RPS6KA3 were significantly elevated in resistant cells (Fig. 7B and Supplementary Fig. 7B to D). Increased phosphorylation sites in RPS6KA4 linked to its activity (Ser^360^ and Ser^687^) [33, 34] also associated with sensitivity to PF-3758309 (Supplementary Fig. 7C). In addition, while no corelation was found between CDK7 activity or phosphorylation and compound response, the phosphorylation of AMPK downstream targets correlated to sensitivity to PF-3758309 (Supplementary Fig. 8).

Next, we asked whether the phosphorylation of sites decreased by PF-3758309 in AML cell lines and primary cells are also determinants of response to the compound. To this end, we used our CTAM algorithm [20] to obtain average phosphorylation scores of phosphorylation sites previously found to be inhibited by the PAKi (Figs. 2, 3, 4, 5 and 6). We found that these CTAM signatures were elevated in sensitive cases, with the CTAM signature obtained from AML primary cells showing the most significant association (*p* = 3.8e-05, Fig. 7C). Within the set of phosphopeptides targeted by PF-3758309 in primary cells, the phosphorylation of PHF2 at Ser^705^, PCNP at Ser^147^, IFI16 at Ser^95^ and ZNF638 at Ser^636^ showed the highest correlation with PF-3758309 sensitivity, while the sites SAFB at Ser^601^ and Ser^604^ and PPHLN1 at Ser^133^ showed the highest correlation with resistance (Fig. 7D).

To test if these phospho-markers are predictive of response in an independent cohort of patient samples, we obtained data from 32 AML patients with poor risk karyotypes (Fig. 7E), for which ex vivo responses to PF-3758309 and phosphoproteomics data are available [22]. We found 36 phosphopeptides significantly correlated with drug response in this dataset, 12 of which overlapped with those identified from the first cohort of patients (Fig. 7F). Of these, PHF2 at Ser^705^ was the most consistently elevated phospho-marker across both datasets (Fig. 7D and E).

In summary, we found that activities of the PF-3758309 targeted kinases PAK1, RPS6KA1 and RPS6KA3 and the phosphorylation of PF-3758309 targeted sites were significantly correlated with the ex vivo response to the compound in primary AML cells. We also identified ten phosphorylation sites targeted by PF-3758309 that predict sensitivity to the compound in two independent cohorts of AML patients.

### Machine learning models predict sensitivity of AML primary blast to treatment with PF-3758309

**Fig. 8:**
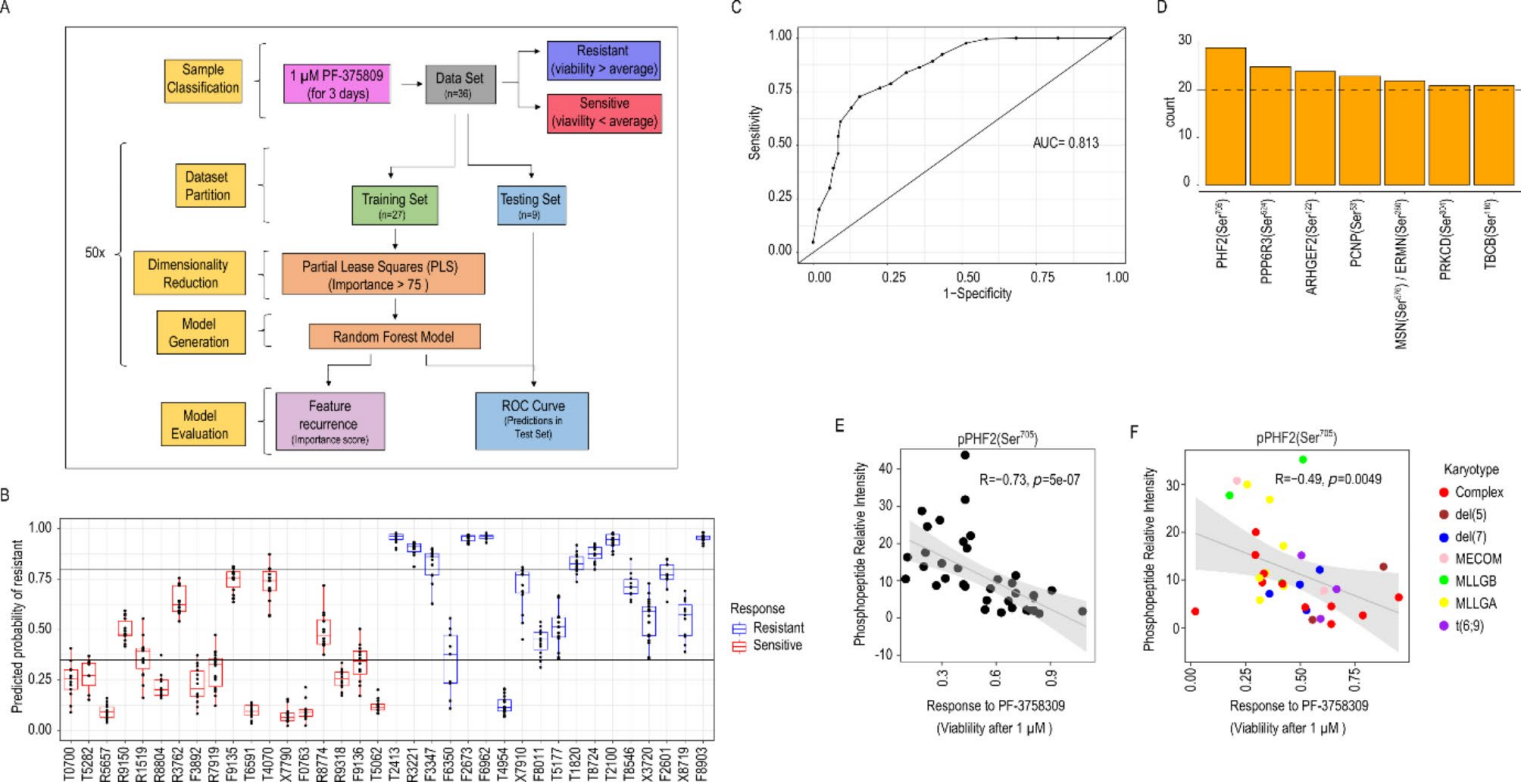
Machine learning classified AML primary samples as sensitive or resistant to PF-3758309 treatment. **A**. Scheme of the workflow used to generate and evaluate the machine learning models. **B**. Predicted probability of being resistant to ex vivo treatment with 1 µM PF-3758309 across AML primary samples when selected for the testing set. **C**. ROC curve showing sensitivity and precision of the classification models across increasing cut off values of predicted probability of being resistant. **D**. Phosphopeptides that most frequently showed importance higher than 70 across ML models. **E**. Correlation between PHF2 phosphorylation and response to PF-3758309 in the cohort from [14]. **F**. Correlation in the cohort from [22]. Correlation was determined using Spearman correlation values

To identify determinants of responses to PF-3758309 in a more untargeted manner and thus complement the approach outline above based on classical statistical learning, we used a modification of a previously described pipeline [19] to generate machine learning (ML) models to classify AML samples as sensitive or resistant (Fig. 8A and methods).

Data points in Fig. 8B show the predicted probability of each primary case of being resistant when it was included in the testing set across the 50 modelling cycles. All samples were included in the testing set in multiple models and, in general, sensitive samples (red data points in Fig. 8B) presented low probabilities of being resistant while those that were resistant (blue in Fig. 8B), showed high probability of resistance (Fig. 8B). ROC analysis showed an area under the curve of 0.81, indicating that our ML models can robustly classify primary AML cells as resistant or sensitive to ex vivo treatment with 1 µM PF-3758309 (Fig. 8C).

To identify sites responsible for model performance, we considered the phosphopeptides that more frequently contributed to model accuracy (importance > 75) (Fig. 8D). Phosphosites PHF2 at Ser^705^, PPPR6R3 at Ser^524^, PCNP at Ser^53^, MSN or ERMN at Ser^576^ or Ser^280^, PRKCD at Ser^304^ and TBCB at Ser^110^ were selected in at least 20 of the 50 ML models (Fig. 8D). Of these, six and one phosphopeptides were increased in sensitive or resistant cases, respectively (Fig. 8E; Supplementary Fig. 9A). Furthermore, the phosphorylation of PHF2 at Ser^705^, ARHGEF2 at Ser^121^ and MSN/ERMN at Ser^576^ or Ser^280^ was reduced after 2 h treatment with 1 µM PF-3758309 in primary blast (Supplementary Fig. 9B). Of note, in an independent dataset of 32 poor risk AML cases [22], the phosphorylation of PHF2 at Ser^705^ was significantly elevated in cases sensitive to ex vivo treatment with 1 µM PF-3758309 (Fig. 8F), thus confirming the relevance of this site as a marker of PAK activity and PAKi sensitivity in an unrelated patient cohort.

Finally, leukemic cells of patients treated with new targeted therapies should be more sensitive to the therapy than healthy blood cells from the same patient. Therefore, we compared the sensitivity to PF-3758309 of leukemic cells from the poor risk cohort and PBMCs from 2 healthy donors. We found that the drug was more efficient in responder cases, separated based on average response, than in healthy blood cells (Supplementary Fig. 10). Thus, these data highlight the need of markers and algorithms that can identify responder cases.

In summary, ML models using phosphoproteomics data accurately classified AML primary samples as sensitive or resistant to ex vivo treatment with 1 µM of PF-3758309 and provided further evidence that phosphorylation of PHF2 at Ser^705^ predicts sensitivity to PF-3758309.

## Discussion

Despite advances in treatment, AML continues to be a disease of unmet clinical need. Pre-clinical studies have shown that PAK isoforms are frequently overactive in AML [11] and PAK inhibitors are effective in reducing the proliferation and viability of AML cell lines and primary cells [6, 12, 14, 35]. However, no PAK inhibitor has been approved for clinical use. We undertook a characterization of PAK inhibitors’ mode of action to identify processes with roles in AML cell viability which could represent new targets for therapeutic intervention.

We decided to focus our study on PF-3758309 because this was the most potent compound in our assays, but we also included in the analysis the PAK inhibitors FRAX-486 and IPA-3 to monitor effects that could be attributed to PAK inhibition.

In addition, the observation that PF-3758309 inhibits cell proliferation in multiple in vivo cell line and patient derived xenograft models [8, 36–43] and that PAK inhibitors are effective in in vivo AML models [6, 44], suggest that PF-3758309 can also be active against AML cells in in vivo systems.

The reduction of autophosphorylation sites in PAK1 and PAK2 validated that all inhibitors targeted PAKs and served as positive controls or our phosphoproteomic experiments. The effects on the phosphorylation patterns differed across inhibitors and cell lines and only a few phosphopeptides were targeted by all inhibitors. Reasons for the heterogeneity of the changes in phosphorylation across the PAK inhibitor treatments could include: (i) PF-3758309 and FRAX-486 are ATP competitors that bind to the ATP binding pocket of PAK while IPA-3 is an allosteric inhibitor that impairs the binding of PAK to its activator. (ii); PF-3758309 is a pan-PAK inhibitor that mainly targets PAK4 while FRAX-486 mainly inhibits Group I PAKs (PAK1, PAK2 and PAK3); (iii) PAK inhibitors present marked differences on their off-target effects.

Our study allowed us to identify multiple kinases and processes targeted by PF-3758309 relevant for AML maintenance and progression. For example, AMPK activity promotes leukaemogenesis and confers metabolic stress resistance to leukaemia initiating cells (LICs). Furthermore, AMPK inhibition synergizes with the metabolic stress caused by dietary restriction [45]. Similarly, CDK7 inhibition supress the growth of GRP56^+^ LICs and CDK7 inhibitors synergize with venetoclax in inhibiting the in vitro and in vivo growth of AML cells [46].

Furthermore, as another process that could be targeted in AML, we found that PF-3758309 reduced the expression of proteins that are part of the ribosome structure (Fig. 3E and Supplementary Fig. 2B). AML progenitor cells express high levels of ribosomal proteins and ribosomal proteins confer selective advantage to malignant cells by regulating translation, proliferation, cellular homeostasis, DNA repair and apoptosis [47]. Therefore, therapies that target the expression of ribosomal proteins could be of interest in the field of AML.

Our study suggested that targeting PAK may be an effective way of reducing c-MYC activity. All PAK inhibitors reduced the phosphorylation of multiple sites in c-MYC including Ser^62^ which increases the stability of the protein by preventing its degradation by the proteasome system [48], concomitant with a reduction in c-MYC protein levels and the expression of its downstream transcriptional targets (Fig. 4C-E). Our data showing that 3 different PAK inhibitors reduced c-MYC phosphorylation together with reports showing that genetic knockdown of PAK1 reduced c-MYC expression [6] suggest that the effect of PF-3758309 on c-MYC can be partially explained by the inhibition of PAK1. However, c-MYC phosphorylations at Thr^58^ and Ser^62^ are not described as direct targets of PAK proteins, and despite several mechanisms by which PAK can regulate c-MYC phosphorylation, expression and activity have been reported [49], PF-3758309 targets multiple kinases that can also regulate c-MYC. Therefore, further experiments are required to clarify if kinases other than PAK, which are also targeted by PF-3758309, have a role in c-MYC phosphorylation, expression and activity. This global transcription factor regulates the expression of 15% of all genes and plays critical roles in preventing apoptosis and promoting drug resistance including [50]. However, c-MYC is a difficult to drug protein and indirect ways to reduce its activity, with for example PAK inhibitors, offer alternatives to target this oncoprotein for therapeutic benefit.

AML subtypes are driven by specific proteins that are frequently used as targets for precision medicine therapies. DOT1L is a methyltransferase that binds to MLLT10 to target lysine 79 of histone H3, and its deregulation drives leukaemogenesis in some AML subtypes [51]. P31/Fuj cells present a translocation between chromosomes 10 and 11 that produces a PCAM-MMLT10 fusion protein that causes the deregulation of DOT1L [26, 52]. DOT1L phosphorylation at Ser^982^ was only detected in P31/Fuj cells and PF-3758309 reduced this site. Clinical trials are evaluating the used of DOT1L inhibitors to treat AML cases with deregulated DOT1L activity. The effect of DOT1L phosphorylation at Ser^982^ is not known but the fact that it is over-phosphorylated in cells that present PICALM-MLLT10 translocations suggest that DOT1L phosphorylation and activity are linked. If this is the case, PF-3758309 treatment could present a novel mechanism to modulate deregulated DOT1L activity.

MV4-11 cells present a FLT3-ITD mutation that renders a hyper-active and hyper-phosphorylated form of the FLT3 tyrosine kinase, which in turns phosphorylates STAT5B at Try^699^ to drive leukaemia progression [28, 53]. STAT5B-Tyr^699^ phosphorylation was detected in MV4-11 cells only, and PF-3758309 reduced the phosphorylation of this site as well as those on FLT3 in this cell line. These observations indicate that PF-3758309 targets FLT3-ITD. Consistent with this conclusion, the FLT3-ITD positive cell lines (MV4-11 and MOLM-13) were the most sensitive to PF-3758309 across a panel of 9 AML cell lines, highlighting the relevance of the off-target effect of PF-3758309 on FLT3-ITD mutant cells.

We found that PF-3758309 reduced cell proliferation and induced apoptosis (Fig. 1) and cell proliferation linked to effects on the cell cycle via a reduction in the activity of the transcription factor E2F1 that plays a crucial role in the G1/S transition [54]. In addition, we observed an increase of the myeloid markers CD11b and CD15 after treatment with PF-3758309 together with an increase in the transcriptional activity of CEBPA, CEBPB and SPI1 (PU.1) and the expression of proteins linked to cell adhesion and receptor signalling. These observations indicate that PF-3758309 also induce myeloid differentiation of AML cells (Figs. 3E and 5B) [55].

In general, the effects that PAK inhibitors had on the phosphoproteome of cell lines were also observed in a set of eight primary cases. However, testing these compounds in primary cases revealed further mechanistic heterogeneity in the biology regulated by targets of PAK inhibitors, which we observed at the molecular and functional levels. Given the variability in responses to the compound, a successful targeted therapy would require the identification of patients more likely to respond in the clinic.

To characterise this mechanistic variability, and identify determinants of response, initially, we took a hypothesis driven approach based on the knowledge obtained from our phosphoproteomics data. Pearson correlation of the ex vivo response to PF-3758309 with the activity, phosphorylation of kinases or proteins targeted by this compound in a set of primary blast from 36 AML cases [14] revealed that the activities of PAK1, RPS6KA1, RPS6KA3 and others are elevated in sensitive primary AML cases (Fig. 7A-C). These observations indicate that the extent of activation of kinases targeted by PF-3758309 determines responses to the PAKi. Importantly, these determinants of response identified by mining a set of 36 cases were validated in an independent cohort of 50 cases with poor risk karyotypes (Fig. 7D-F).

To complement the knowledge-based analysis, we also used an unbiased approach based on random forest. The developed models were able to accurately classify the AML primary samples as sensitive or resistant to ex vivo treatment with PF-3758309. Interestingly, both the ML and the statistical correlation approach identified the phosphorylation of the histone demethylase PHF2 at Ser^705^ as the best marker of sensitivity to PF-3758309 (Fig. 8D). The observation that the phosphorylation of PHF2 at Ser^705^ also correlated with sensitivity to PF-3758309 in a completely independent cohort of AML patients highly reinforces the value of this phosphorylation as a predictive marker. However, we acknowledge that the predictive value of this marker will need to be further studied in larger cohorts and ultimately in prospective clinical trials. PHF2 main substrates are histones H3 at K9 and H4 at K20 as well as other proteins [56–58]. PHF2 regulates DNA damage response and genomic integrity and participates in the inflammatory response [57, 59]. The role of PHF2 in cancer is controversial since its expression is increased in oesophageal and renal carcinoma, but the *PHF2* gene is mutated in gastric and colorectal cancer as well as deleted or hypermethylated at the promotor region in breast cancer [60–63]. PKB and AMPK phosphorylation of PHF2 at multiple sites promotes the activity of the demethylase but the effect of its phosphorylation at Ser^705^ is not known [58, 64]. PF-3758309 reduces PHF2 phosphorylation at Ser^705^, suggesting that the ability of PHF2 to promote resistance or increase sensitivity is directly targeted by the inhibitor.

## Conclusions

In summary, PAK inhibitors target numerous kinases and other proteins with roles in the maintenance and progression of AML and provide a means to inhibit key pro-proliferative processes in different AML subtypes. These include FLT3 in FLT3-ITD positive cells, DOT1L in PICALM-MLLT10, and the oncogene c-MYC, which is overactive in most cancer types. Approaches based on classical statistics and ML identified PHF2 phosphorylation at Ser^705^ as potential response biomarker. Our data and research approach could contribute to the identification of response biomarkers and the implementation of precision medicine in AML using multi-targeted kinase inhibitors.

## Electronic supplementary material

Below is the link to the electronic supplementary material.

Supplementary Material 1

Supplementary Material 2

Supplementary Material 3

## Data Availability

Mass spectrometry data generated in this manuscript have been deposited to the ProteomeXchange Consortium via the PRIDE partner repository [62] with the dataset identifier PXD056514 and 10.6019/PXD056514. Phosphoproteomics data on AML primary cells used to generate the ML models and assess correlation with drug response data were generated in [11, 22] and can be downloaded from the PRIDE repository with the dataset identifiers PXD005978 (DOI 10.6019/PXD005978) and PXD032980 (DOI 10.6019/PXD032980).

## Abbreviations

AML: acute myeloid leukaemia
AMPK: 5’ AMP-activated protein kinase
c-MYC: cellular-myelocytomatosis
CAMK: calmodulin kinase
CD: cluster of differentiation
CDK: cyclin dependent kinase
CHEK2: checkpoint kinase 2
CLK1: CDC2-like 1
CTAM: compound-target activity marker
DNAPK: DNA protein kinase
DMSO: dimethyl sulfoxide
DOT1L: disruptor of telomeric silencing 1-like
FLT3-ITD: fms-like tyrosine kinase 3– internal tandem duplication
HAG: human gamma-globulins
KSEA: kinase substrate enrichment analysis
MAPK: mitogen-activated protein kinase
ML: machine learning
MLLT10: mix-lineage leukaemia translocated to 10
PAKs: P21-activated kinases
PCALM: Phosphatidylinositol binding clathrin assembly protein
PDTs: putative downstream targets
PDX: patient derived xenograft
PHF2: PHD finger protein 2
PHKG2: phosphorylase kinase catalytic subunit gamma 2
PKC: protein kinase C
STAT5: signal transducer and activator of transcription 5
RS6PKs: ribosomal S6 protein kinases
SYK: spleen tyrosine kinase
XIC: extracted ion chromatogram
