# Supplementary material for: Phosphoproteomics identifies determinants of PAK inhibitor sensitivity in leukaemia cells
Source: Cell Commun Signal. 2025 Mar 13;23:135. doi: 10.1186/s12964-025-02107-0 (PMC11907924; doi:10.1186/s12964-025-02107-0)
Supplement: Supplementary file 3 — Supplementary Material 3 [file 12964_2025_2107_MOESM3_ESM.docx]

**Supplementary Figure Legends**

**
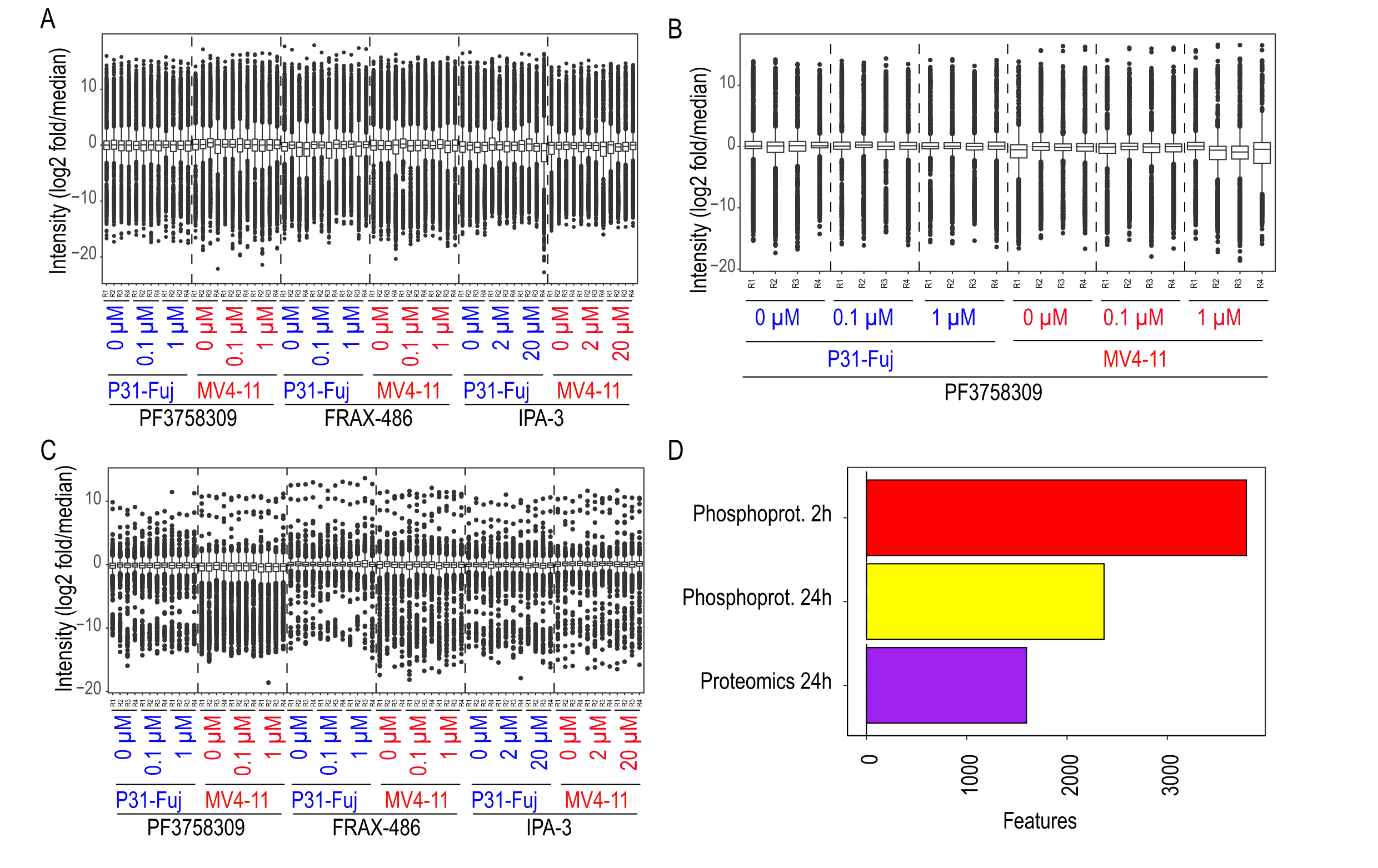
**

**Supplementary Figure 1. Quality control for the phosphoproteomics and proteomics studies**. Box plots of the peptide intensities for each LC-MS/MS run in the 2h phosphoproteomics (**A**), 24h phosphoproteomics (**B**), and 24h proteomics (**C**) experiments. **D**. Number of phosphopeptides or proteins identified and quantified in each experiment.

**
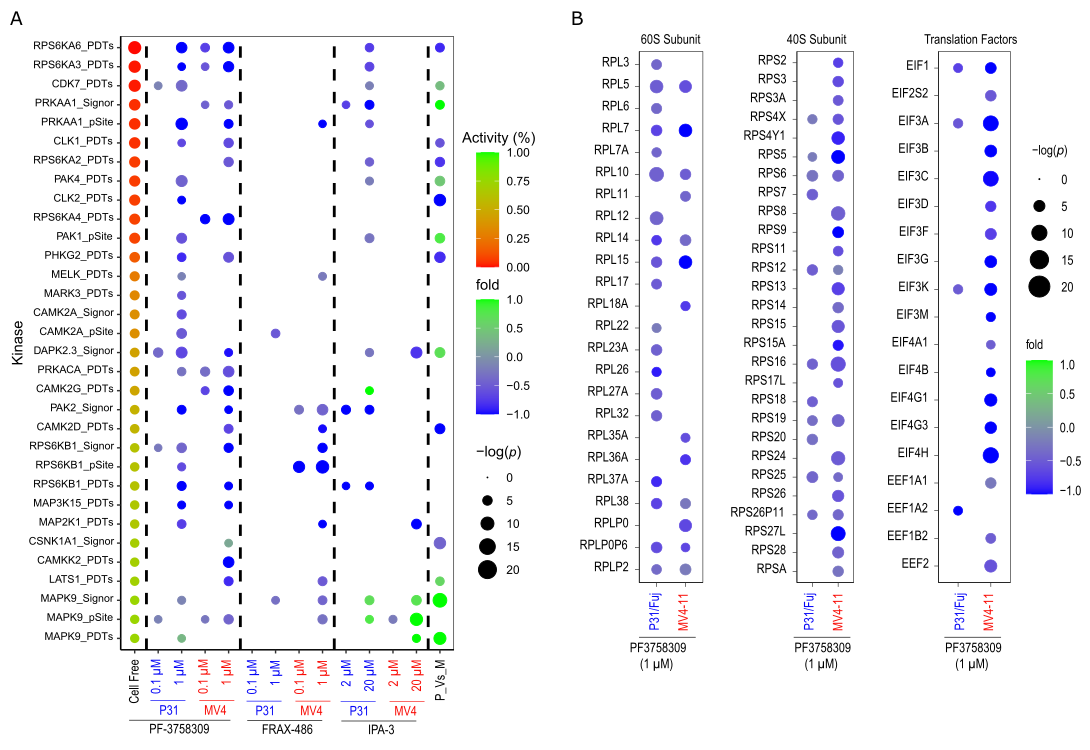
**

**Supplementary Figure 2. PF-3758309 inhibited PAK activities more efficiently than FRAX-486 and IPA-3 and downregulated the expression of ribosomal proteins and translation factors. A.** Dot plot showing kinase activities affected by 2h treatment with the indicated PAKi at the indicated concentrations. Changes in kinase activity were estimated using KSEA. **B.** Dot plot showing ribosomal proteins and translation factors downregulated after 24h treatment with 1 µM PF-3758309. Changes in protein expression were measured using label free proteomics. Statistical differences were assessed using t-test (n=4 independent replicates). All changes shown presented a *p* ≤ 0.05.

**
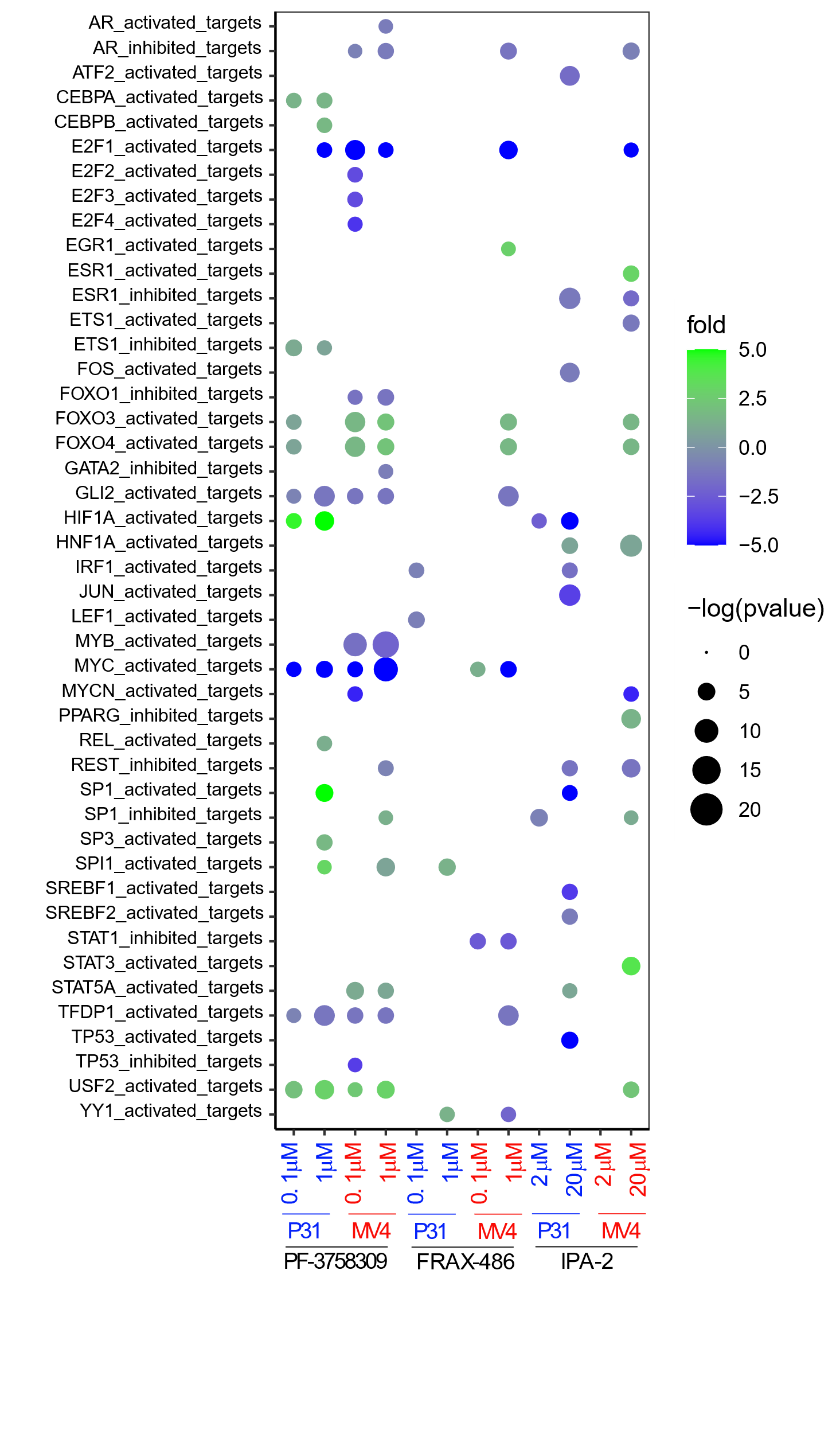
**

**Supplementary Figure 3. PF-3758309 decreased the activity of c-MYC and E2F1 and increased the activity of SP1 in P31/Fuj and MV4-11 cells.** Proteomics data on P31/Fuj and MV4-11 cells treated with 0.1 or 1 µM of PF-3758309 or FRAX-486 or 2 or 20 µM of IPA-2 for 24h were used to estimate transcription factor activity. Statistical differences were calculated using unpaired Student’s t-test (n=4).

**
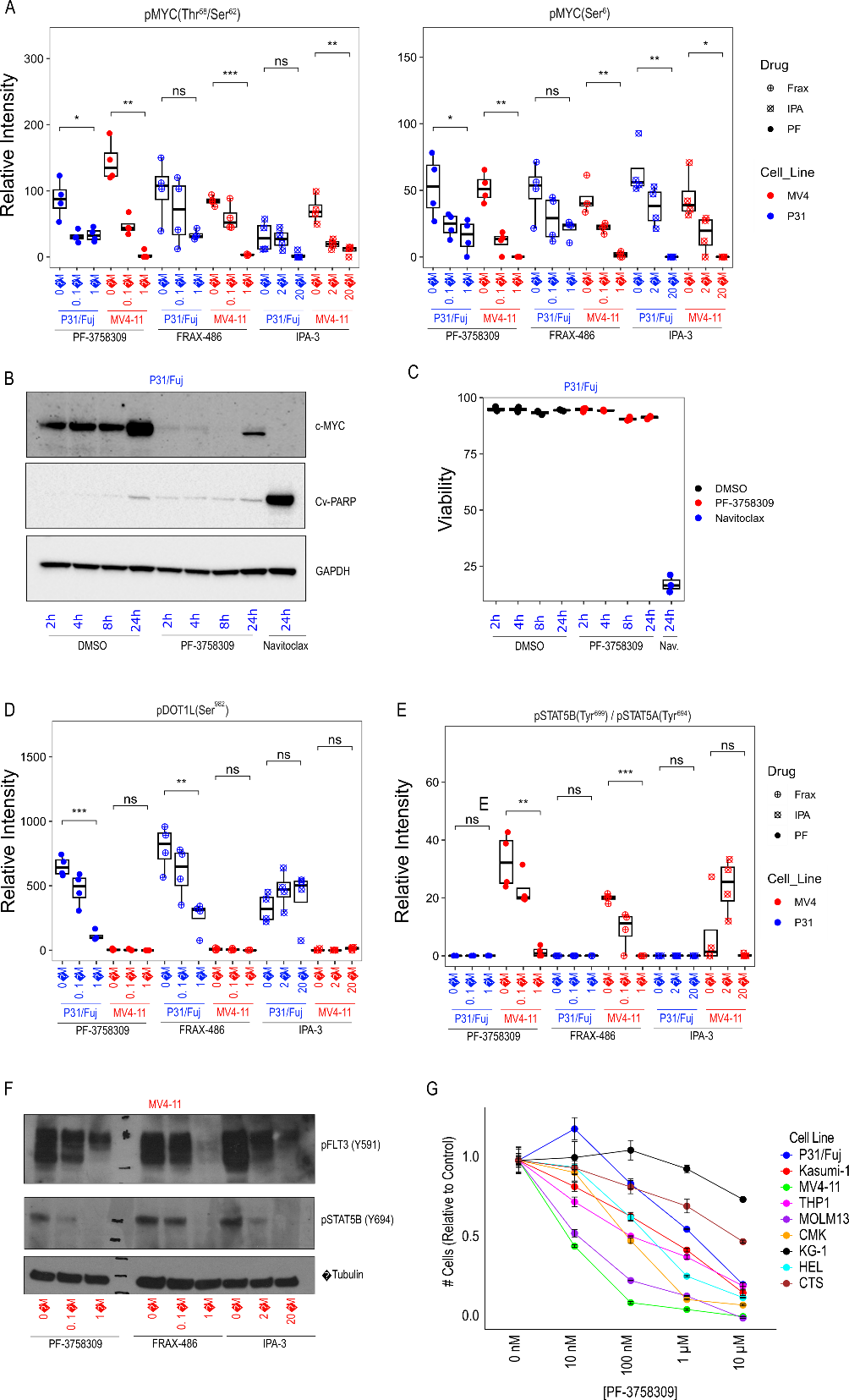
**

**Supplementary Figure 4. PAKi reduced the phosphorylation of c-MYC, DOT1L, STAT5B and FLT3, and cells positive for FLT3-ITD are more sensitive to PF-3758309. A.** The PAKi PF-3758309, FRAX-486 and IPA-3 reduced the phosphorylation of c-MYC at Thr^58^, Ser^62^ and Ser^6^ after 2h treatment. **B.** The reduction of c-MYC induced by 1 µM treatment with PF-3758309 is not associated to apoptosis. A 24h treatment with 10 µM navitoclax was used as positive control for apoptosis. Apoptosis was monitored with the apoptotic marker cleaved PARP1 (Cv-PARP). **C.** Treatment with 1 µM PF-3758309 does not induced cell death after 24h or earlier. Cell dead in P31/Fuj cells was measured using Guava ViaCount. The PAKi PF-3758309, FRAX-486 and IPA-3 reduced the phosphorylation of DOT1L at Ser^982^ (**D**) and STAT5B at Tyr^694^ (**E**) after 2h treatment. Statistical differences were calculated using unpaired two tailed t-test (n=4). **F**. Western blot showing that PAKi reduced the phosphorylation of FLT3 at Tyr^591^ and STAT5B at Tyr^694^ after 24h treatment in MV4-11 cells. **G.** The FLT3-ITD positive cells MV4-11 and MOLM-13 are more sensitive to PF-3758309 than FLT3 WT cells lines. The indicated cell lines were treated with the indicated concentrations of PF-3758309 for 72h and cells were counted using Guava ViaCount (n=4).

**
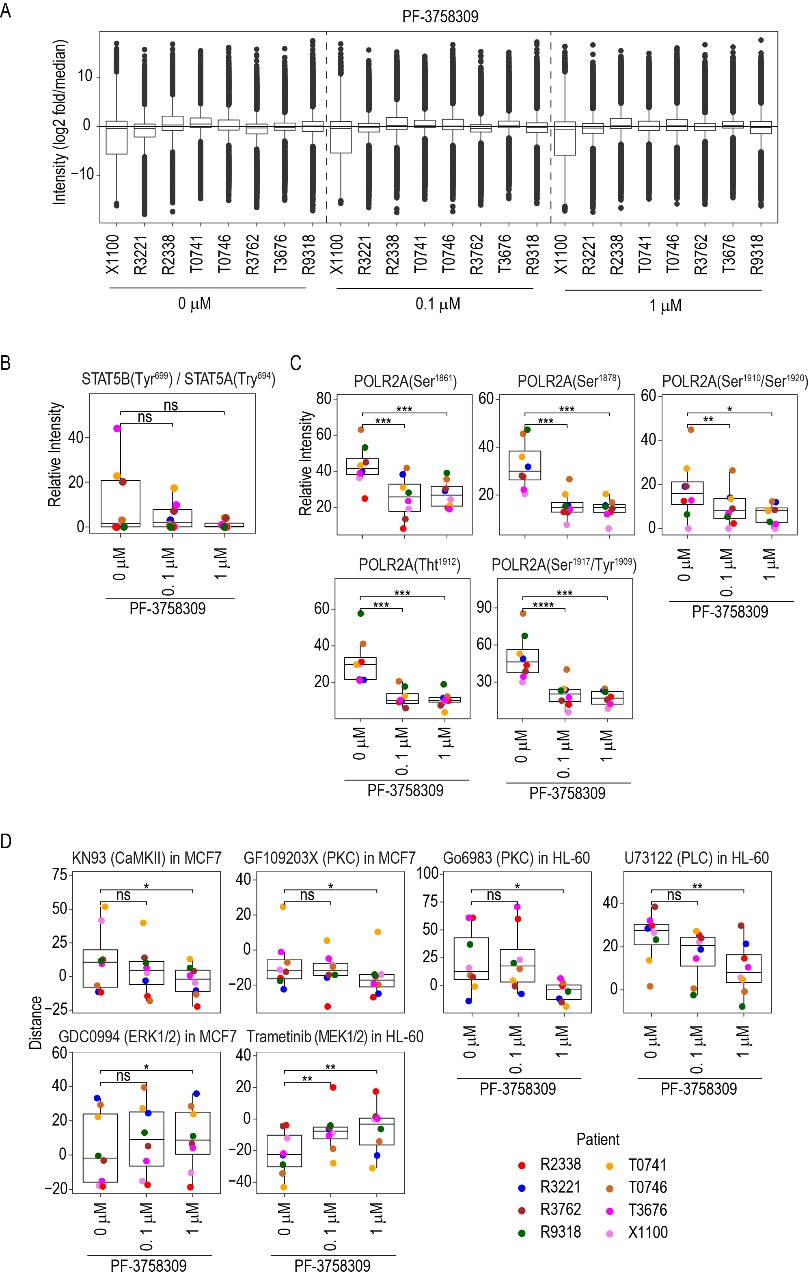
**

**Supplementary Figure 5. PF-3758309 downregulated the phosphorylation of STAT5, RNApolII and peptides downregulated by CAMK, PCK, and PLC inhibitors in AML primary cells.** AML primary cells were treated with 1 µM PF-3758309 for 2h and processed for phosphoproteomics analysis. **A.** Box plots of the peptide intensities for each LC-MS/MS run. **B.** PF-3758309 reduced the phosphorylation of STAT5 at Tyr^694^ when detected (**B**), RNApolII (**C**) and peptides downregulated by KK93 (CAMKi), GF109203X and Go6983 (PCKi), and U73122 (PLCi) (**D**). PF-3758309 increased the phosphorylation of peptides downregulated by GDC0994 (ERK1/2i) and trametinib (MEKi) (**D**). Statistical differences were determined using paired Student’s t-test (n=8). **** p ≤ 0.0001, *** p ≤ 0.001, ** p ≤ 0.01 and * p ≤ 0.05.


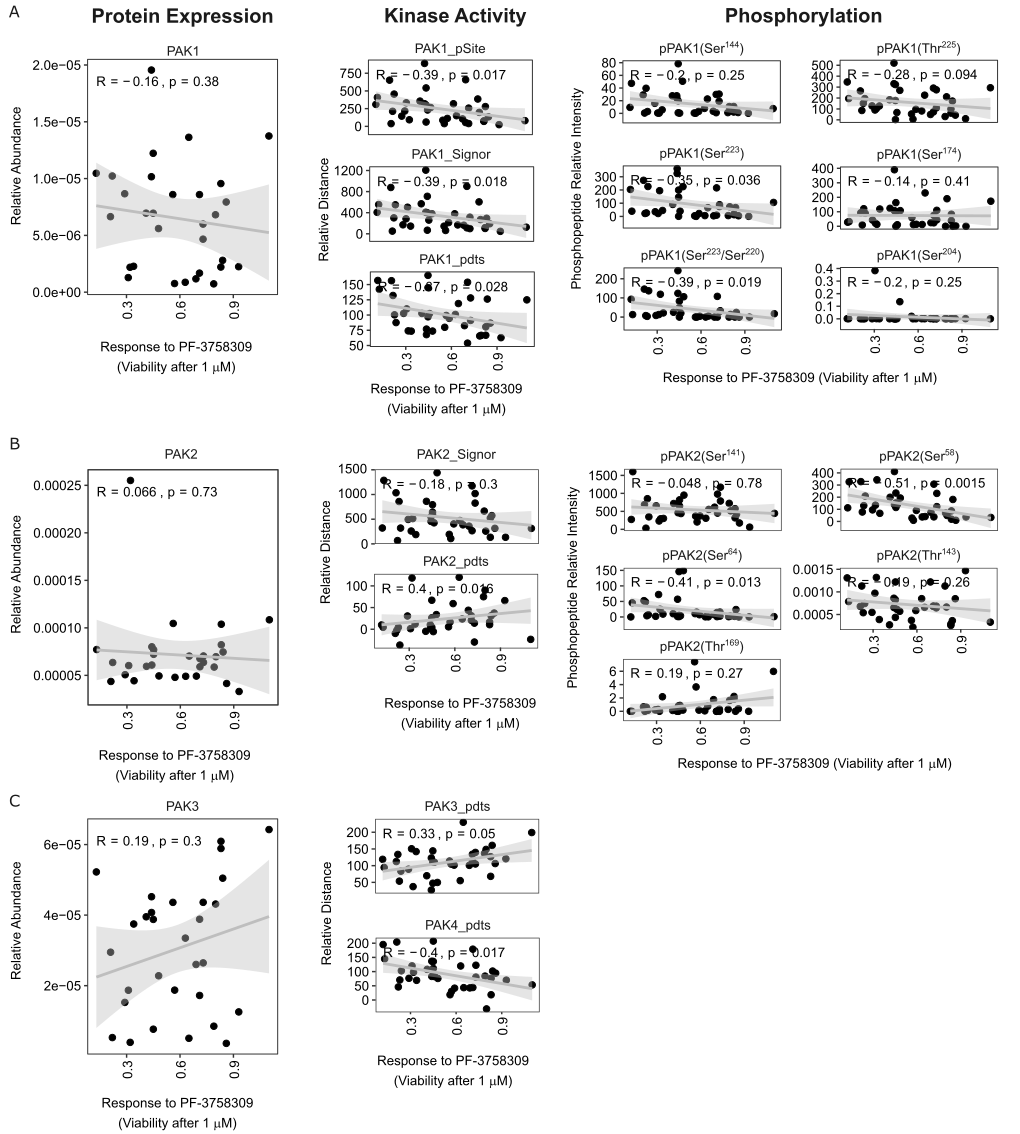


**Supplementary Figure 6. Sensitivity to PF-3758309 correlates with PAK1 activity and phosphorylation in AML primary cells.** **A** Correlation between sensitivity to PF-3758309 and the expression, phosphorylation and activity of PAK1. **B** Correlation between sensitivity to PF-3758309 and the expression, phosphorylation and activity of PAK2. **C** Correlation between sensitivity to PF-3758309 and the expression and activity of PAK3 and 4. Correlation was determined using Spearman correlation values.


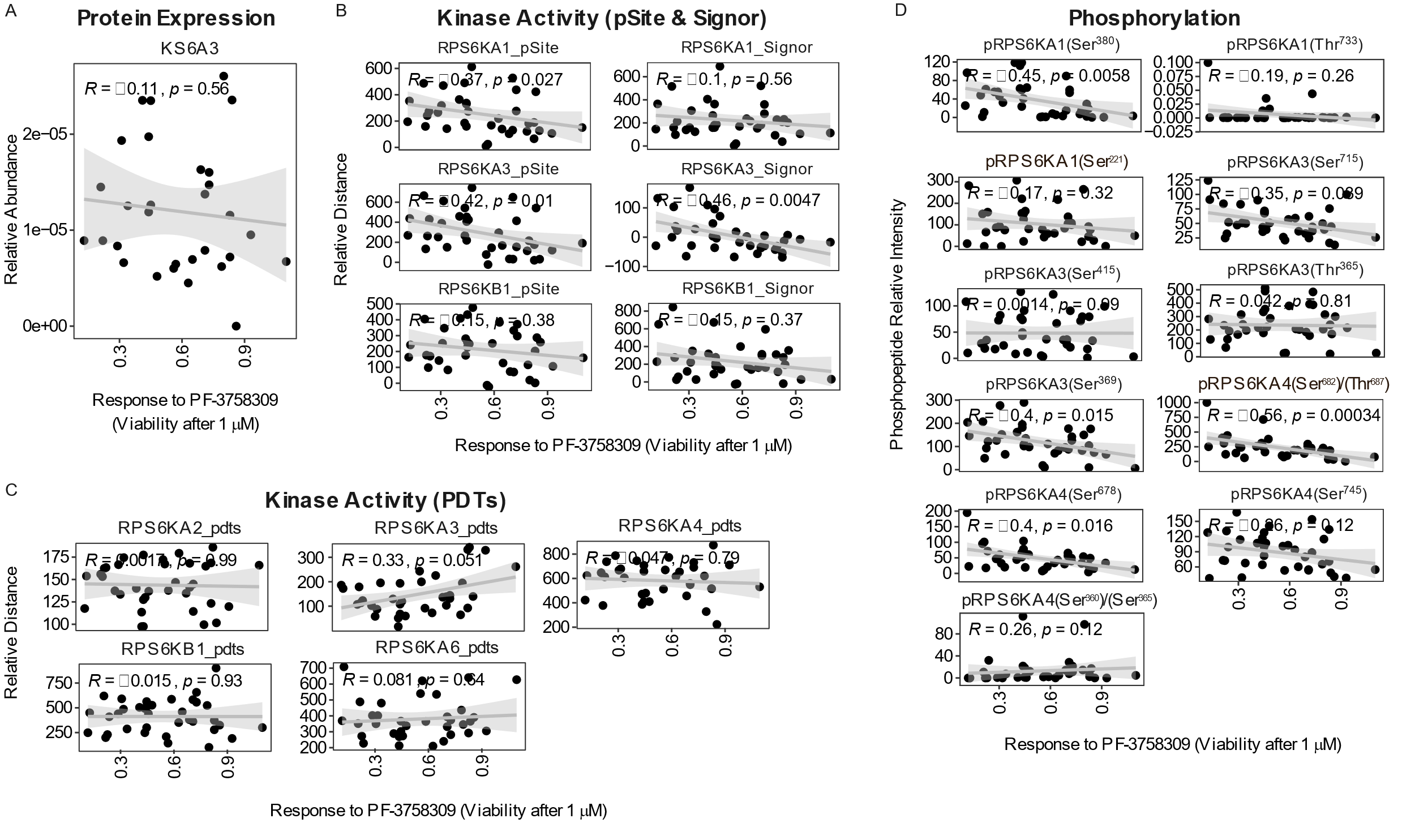


**Supplementary Figure 7. Sensitivity to PF-3758309 correlates with RPS6KA1 and 3 activity and phosphorylation in AML primary cells.** **A.** Correlation between sensitivity to PF-3758309 and RPS6KA3 protein expression. **B.** Correlation between sensitivity to PF-3758309 and RPS6KA1, RPS6KA3 and RPS6KB1 activity measured using the pSite and Signor databases. **C.** Correlation between sensitivity to PF-3758309 and RPS6KA2, RPS6KA3, RPS6KA6 and RPS6KB1 activity measured using the PDTs database. **D.** Correlation between sensitivity to PF-3758309 and the phosphorylation of RPS6KA1, RPS6KA3 and RPS6K4. Correlation was determined using Spearman correlation values.


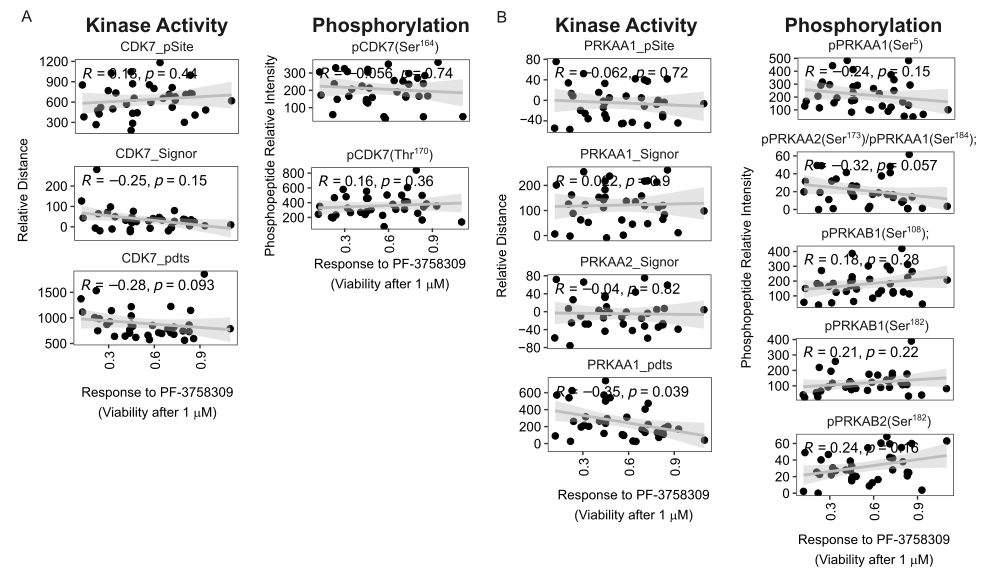
**Supplementary Figure 8. Sensitivity to PF-3758309 does not clearly correlate with CDK7 or AMPK (PRKAA) activity or phosphorylation in AML primary cells. A.** Correlation between sensitivity to PF-3758309 and CDK7 activity or phosphorylation. **B.** Correlation between sensitivity to PF-3758309 and PRKAA or PRKAB activity or phosphorylation. Correlation was determined using Spearman correlation values.


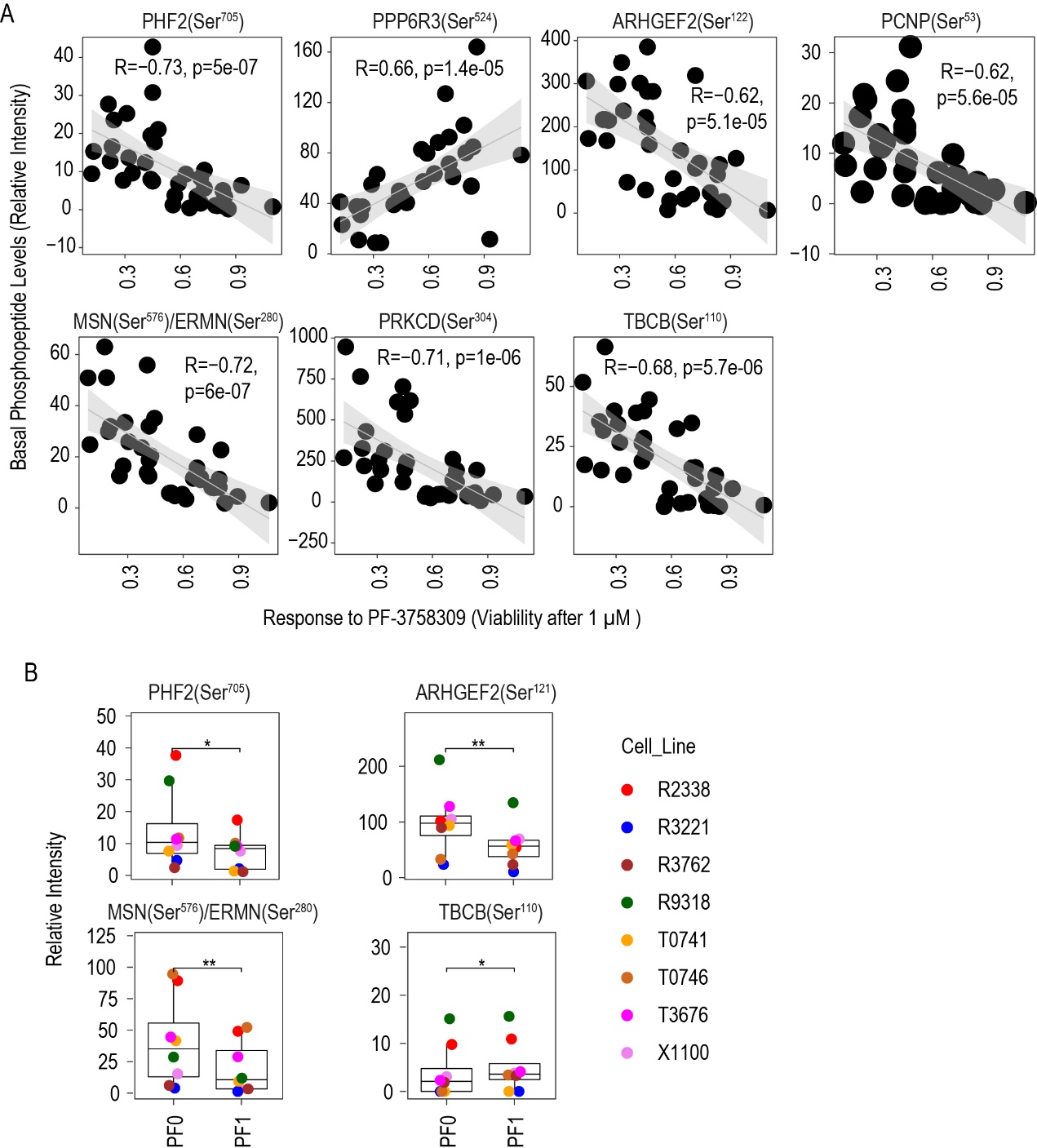


**Supplementary Figure 9. Phosphopeptides relevant for the ML models showed in Figure 7 that correlate with ex-vivo response to PF-3758309 treatment and are affected by PF-3758309 treatment in AML primary cells. A.** Correlation between the phosphorylation of the indicated phosphopeptides and the *ex-vivo* response to 1 µM PF-3758309 treatment for 72 h in AML primary cells. **B**. Effect of 1 µM treatment with PF-3758309 for 2h in the phosphorylation of the sites showed in **A**. Correlation in A was determined using spearman coefficient. Statistical differences in B were calculated using paired Student’s t-test (n=8). **** *p* ≤ 0.0001, *** *p* ≤ 0.001, ** *p* ≤ 0.01 and * *p* ≤ 0.05.


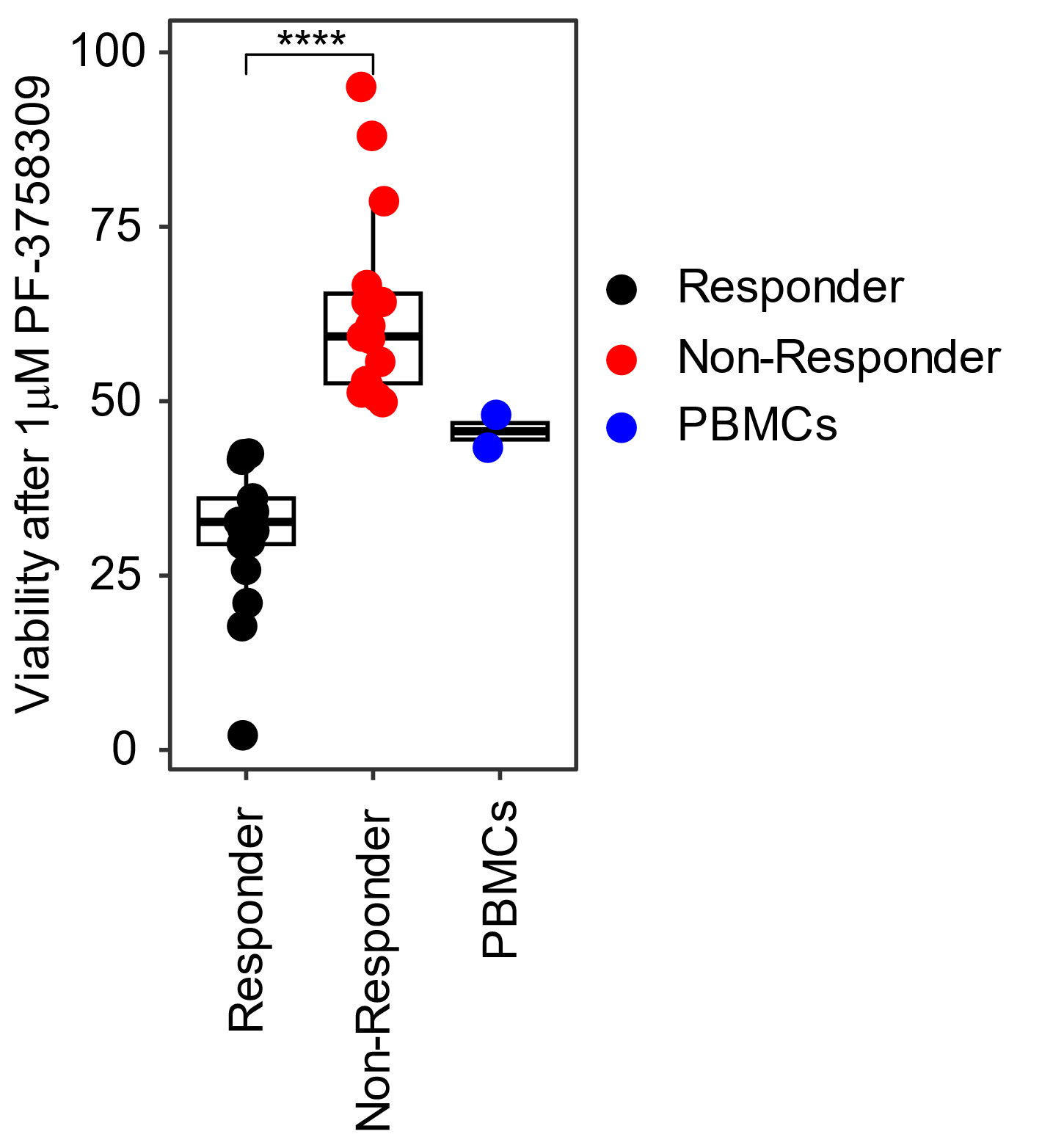


**Supplementary Figure 10. PF-3758309 is more effective in responder leukemic cells than in healthy blood cells.** Mononuclear cells from AML patients and from the peripheral blood of healthy individuals (PBMCs) were treated ex vivo with 1 mM PF-3758309 for 72h. AML samples were separated into responders or non-responders to the drug based on the average response. Statistical differences were determined using paired Student’s t-test. **** p ≤ 0.0001.
